# Patterns of ties in problem-solving networks and their dynamic properties

**DOI:** 10.1038/s41598-020-75221-3

**Published:** 2020-10-22

**Authors:** Dan Braha

**Affiliations:** 1 New England Complex Systems Institute, Cambridge, MA 02139 USA; 2 University of Massachusetts Dartmouth, Dartmouth, MA 02747-2300 USA

**Keywords:** Computational biology and bioinformatics, Ecology, Psychology, Systems biology, Ecology, Engineering, Mathematics and computing, Physics

## Abstract

Understanding the functions carried out by network subgraphs is important to revealing the organizing principles of diverse complex networks. Here, we study this question in the context of collaborative problem-solving, which is central to a variety of domains from engineering and medicine to economics and social planning. We analyze the frequency of all three- and four-node subgraphs in diverse real problem-solving networks. The results reveal a strong association between a dynamic property of network subgraphs—synchronizability—and the frequency and significance of these subgraphs in problem-solving networks. In particular, we show that highly-synchronizable subgraphs are overrepresented in the networks, while poorly-synchronizable subgraphs are underrepresented, suggesting that dynamical properties affect their prevalence, and thus the global structure of networks. We propose the possibility that selective pressures that favor more synchronizable subgraphs could account for their abundance in problem-solving networks. The empirical results also show that unrelated problem-solving networks display very similar local network structure, implying that network subgraphs could represent organizational routines that enable better coordination and control of problem-solving activities. The findings could also have potential implications in understanding the functionality of network subgraphs in other information-processing networks, including biological and social networks.

## Introduction

Problem-solving is a natural and ubiquitous human activity^1,2^, and is concerned with devising courses of action aimed at changing existing situations into preferred ones^3^. In this sense, the problem-solving activity is key to a variety of fields from engineering and medicine to economics and social planning. Some authors even argue for a direct resemblance between problem-solving processes and the structure of the scientific method^4^.

As problem-solving becomes complex and dynamic, the limited ability of humans to handle complexity and large amount of information is accounted for by decomposing the complex system into components that are relatively independent^1–3^. The problem is then solved collectively by multiple groups (a “group” can include one or more individuals), each solving problems with some degree of independence of others^1–3,5,6^. In such a coordinated problem-solving environment, the decentralized groups make decisions on the basis of information that is available to them locally via the network of interactions with other groups. Often, in such a network of interactions, decentralized groups make decisions even though the local information is not available or known with certainty^2^. In this case, decentralized groups need to make assumptions about the information generated by other units, and use these assumptions in their decisions. Groups update their decisions due to the availability of new information generated by other groups, such as changes in input, updates of shared assumptions, components, boundaries, or the discovery of errors^2^. This interdependence between the various groups makes collaborative problem-solving fundamentally iterative^2,5^ in the sense that as new information becomes available, the decentralized decisions are repeated to come closer to the problem-solving goals or specifications. This iterative problem-solving process proceeds until convergence occurs^2,5^.

One way of thinking about coordinated problem-solving is to view it as involving both exploratory and exploitative behaviors. By receiving information locally via the network of interactions, decentralized groups gather information (exploration) that will ultimately be valuable in discovering a problem solution (exploitation). The tradeoff between the exploration of new possibilities and the exploitation of old certainties^6–8^ is a central concern for a wide range of adaptive processes^9–18^. Gathering too much information via the network of interactions is likely to increase the quality of the over-all coordinated activity at the cost of slower convergence rates. On the other hand, carrying out exploitation more rapidly than exploration, e.g., by limiting the explorative information-gathering via local interactions, is likely to result in rapid convergence of the coordinated activity at the cost of lacking the ability to adapt to significant changes in information, requirements or constraints mediated by connected groups in the network. The latter might result in a suboptimal overall solution. Thus, it is reasonable to expect that the structure—both global and local—of the network of group interactions represents a balance between exploration and exploitation. Another useful view of coordinated problem-solving is to look at it as a social process involving cooperation among self-oriented individuals or groups^4,19^. According to this view, self-oriented individuals or groups cooperate and coordinate with each other, despite being driven to make local decisions that aim to achieve their local goals and viewpoints. This perspective is closely related to understanding the emergence of cooperation among selfish individuals—a key problem in biology^20,21^, network science^22,23^, and the social sciences^24^. In decentralized problem-solving, cooperation among groups is promoted by several communication mechanisms including the creation of shared meaning, high-level goals and views, and common knowledge^4^. Similar to exploration and exploitation, it is likely that the structure of ties in problem-solving networks captures the tension between cooperation and competition, and might account for the long-term cooperative actions between self-oriented groups.

Following the above discussion, it is important to understand how the output dynamics of one group can be affected by changes in the dynamics of other groups via the network of interactions. An important step in this direction is graph-based representations of real-world problem-solving. Steward^25^ applied a square-matrix format (the adjacency matrix of a network) to represent a network of engineering task interactions, and used the method to determine a logical sequence for the tasks being modeled. This method was elaborated and extended in a variety of ways (e.g., using clustering analysis), including applications to a wide range of technical domains^5,26–29^. Braha and Bar-Yam^1,2,30^ and Braha^31^ used complex network theory to identify global statistical features that are shared across a variety of large-scale, real-world engineering problem-solving networks (ranging in size from 120 to 889 nodes), highlighting the similarity between problem-solving networks and other complex networks that occur in nature and society^32^. These global statistical features include sparseness of connections, the “small-world” property characterized by highly clustered nodes and short distance between any two nodes, uneven node degree distributions characterized by a few very highly connected nodes (critical nodes or ‘hubs’), asymmetry between the distributions of incoming and outgoing information flows, disassortativity among nodes, community structure (modularity), and hierarchical network organization^1,2,30,31^.

As discussed above, although the network structure of collaborative problem-solving is static the nodes (subproblems/tasks) represent values that change in time. Understanding the interplay between network structures and the global dynamics and performance of real-world problem-solving networks has been investigated by several researchers. Empirical work on small groups by organizational scientists show a mixed relationship between network density and performance^33–38^. A meta-analysis^34^ of 37 studies of teams (ranging in size from 3 to 15 members) showed a modest positive relationship between team’s performance and network structure such as density of ties and the centrality of team’s leaders in the network. Other studies found an inversely U-shaped relationship between density of ties and team performance^35–37^, while other studies found no association at all^38^. Several laboratory-based experiments (such as graph-coloring tasks) examined the effect of density, clustering, and efficiency (measured in terms of average path length) on the balance between problem-solving exploration and exploitation^18,39–43^. For example, it was suggested that clustering inhibits exploration of new solutions (e.g., by copying and refining solutions of others) and promotes exploration of new knowledge and facts^18^. Research that examines the relationship between structure and performance also benefited from simulations and agent-based modeling^6,44,45^. This research found, for example, that efficient network structures (e.g., fully connected networks) that facilitate fast diffusion of information quickly converge on solutions that are better than the ones corresponding to inefficient networks. However, in the long run, inefficient networks that facilitate exploration of new information could perform significantly better than inefficient networks^6^.

By integrating theory, computational modeling, and empirical data of large-scale problem-solving networks, Braha and Bar-Yam^1,2,30^ provided explanations for how the various organizing principles observed in real-world problem-solving networks affect overall system dynamics. Sparseness and small-world properties were explained in terms of efficiency, exploration, and integration. The average connectivity and the extent of degree correlations in the network determine whether collaborative problem-solving converge on an equilibrium, and how rapidly decentralized groups synchronize their activity. In particular, positively correlated networks tend to slow synchronization and convergence to an equilibrium. The right-skewed degree distributions and the characteristic feature of ‘hubs’ (highly connected nodes) lead to ultra-robustness under the circumstances of adverse fluctuations that affect randomly selected nodes. On the other hand, the right-skewed distributions also make problem-solving networks more fragile and vulnerable to adverse fluctuations that occur at highly connected nodes; a condition that slows synchronization and convergence. At the same time, the right-skewed distributions enable remarkable improvement of synchronization performance when resources are preferentially allocated to the highly connected nodes in the network.

Although global topological features (such as path lengths and degree distributions) provide important insight into problem-solving networks^1,2^, a more refined analysis of repeated patterns of ties (subgraphs) in problem-solving networks is needed to truly understand their large-scale dynamical properties. The frequency of a subgraph with a particular arrangement of ties (typically a subgraph of three or four nodes) in a particular network is the number of different matches of this subgraph, where topologically identical arrangements are counted as the same type of subgraph. Network motifs are defined as subgraphs that are overrepresented in the real-world network relative to their appearance in an ensemble of appropriately randomized networks^46–48^. Identifying subgraphs that are underrepresented in the real-world network (anti-motifs) is of equal importance in characterizing the network structure. The same network motifs may appear in diverse networks^46,49^, suggesting that motifs can delineate broad families of networks^47^, each family is characterized by common basic functionalities (e.g., networks that process information are distinct from networks that process energy flow and transfer). The exploration of subgraphs with two nodes (dyads) and three nodes (triads) has a long and rich history^50,51^, beginning with the work of Holland and Leinhardt^52^ who used three-node subgraphs to study social networks. Similar techniques were applied in a variety of fields, including ecology^53–55^, systems biology^48,49^, economics and finance^56,57^, and neuroscience^58^. The dynamic and functional properties of network motifs in biological and ecological networks were explored both analytically and empirically^48,49,59^. Significant effort has shown that abundant motifs in diverse transcription networks (both sensory and developmental) perform a variety of regulatory and information processing functions, such as balancing homeostasis and plasticity^59^. It was also argued that the patterns of ties of biological and ecological network motifs make them more locally stable—roughly speaking, the tendency for system perturbations to damp out, returning the system to some persistent equilibrium^60^. The stability analysis applied in these works is based on calculating the eigenvalues of randomly generated matrices (representing the Jacobian of an underlying dynamical system)—an approach introduced by Gardner and Ashbey^61^, and extended to study ecological stability^53,60^. For example, Pimm^53^ showed that subgraphs that are commonly found in real ecological food webs (including subgraphs of three, or four species) also tend to be more locally stable. Similar concepts were applied to several biological networks (including transcription, signal transduction, and neuronal networks) where it was shown that the stability properties of all structurally distinct three- or four-node subgraphs are highly correlated with their abundance in the network^49^. The approach presented in the current paper follows this direction of comparing subgraph abundance in real networks with their dynamic properties.

In this paper, we extend our previous work on complex problem-solving networks^1,2,30^ by analyzing the frequency of all three- and four-node subgraphs in diverse real problem-solving networks. We attempt to answer the pertinent question of what determines the frequency of network subgraphs in real problem-solving networks. It has been shown that the system-level structure of many complex systems is best approximated by a hierarchical network organization with seamlessly nested modularity^62^, a property also observed in problem-solving networks^30^. A nested hierarchical organization of problem-solving networks means that there are many highly integrated small groups of individuals, which assemble into a few larger groups, which in turn can be integrated into even larger groups. It is plausible to reason that rapid and effective synchronization of the problem-solving activity evolves by the accumulation of rapidly synchronized intermediate configurations, which are interconnected to form more synchronized complex structures. We thus hypothesize that real problem-solving networks will be biased towards repeated patterns of ties in which it is easier to obtain problem-solving synchronization. In this paper, we show that network subgraphs embedded in a variety of real problem-solving networks can emerge based on such considerations. In particular, we find a high correlation between a dynamic property of a network subgraph—synchronizability—and its frequency and statistical significance in real problem-solving networks. In this paper, synchronizability is characterized as the probability of rapidly coordinating the problem-solving activities, and is determined for each three- and four-node subgraph based on a corresponding dynamical model of collective problem-solving. The results in this paper show that highly-synchronizable subgraphs are overrepresented in the real problem-solving networks, while poorly-synchronizable subgraphs are underrepresented, suggesting that the dynamical properties of subgraphs affect their prevalence, and thus the global structure of problem-solving networks.

## Modeling setup

**Figure 1:**
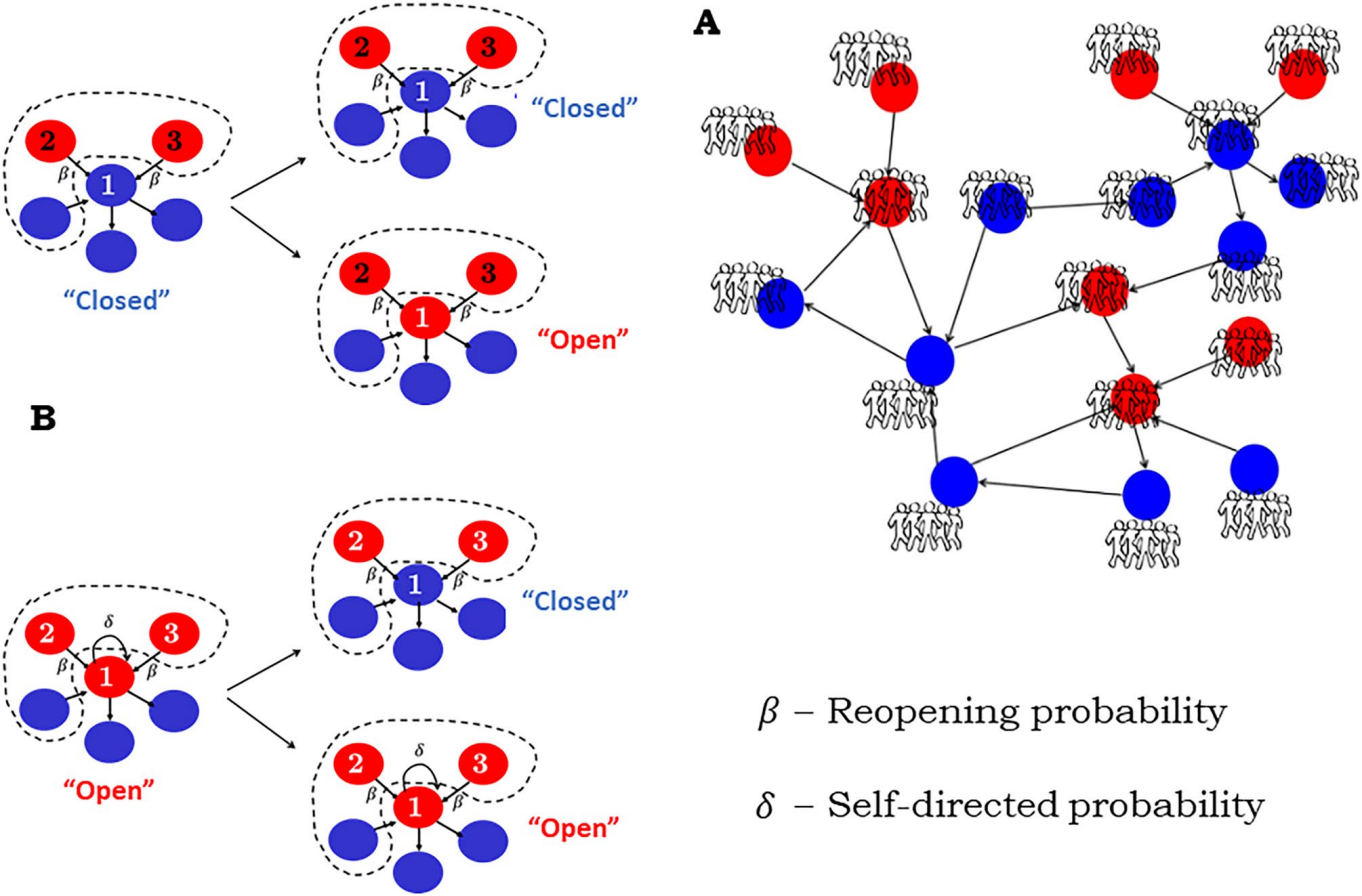
A dynamic network model of collective problem-solving. (**A**) The problem-solving network consists of nodes, representing subproblems attempted by decentralized groups. The groups interact with one another via directed communication links. In the diagram, blue and red nodes represent ‘closed’ and ‘open’ subproblems, respectively. (**B**) The stochastic rules that govern the dynamics of the network. The model involves two parameters—the reopening probability $\upbeta$, and the self-directed probability $\updelta$.

Before studying the dynamic properties of subgraphs, we need to develop a generic dynamical model of collective problem-solving. To this end, we use the well-characterized stochastic model presented in^2^. Consider the scenario of solving a complex problem, which involves a large number of decentralized groups each of which solves a simpler subproblem task. As shown in Fig. 1A, subproblem tasks are represented as the nodes of a directed network, and a directed link from one subproblem to another represents the information dependency between the two subproblems. Each node in the network can be in two states: ‘open’ (if the subproblem is ‘unresolved’) or ‘closed’ (if the subproblem is ‘resolved’). At each time step, a node is selected at random. If the node is in a ‘closed’ state (Fig. 1B, top), its state can be changed depending on the number of ‘open’ nodes connected to it through incoming links. These ‘open’ nodes send out new information that might lead to the reopening of a neighboring ‘closed’ subproblem. More specifically, each ‘open’ subproblem causes a connected ‘closed’ subproblem to reopen its state with a reopening probability $\upbeta$. The strength of this reopening probability $\upbeta$ plays an important role in determining the synchronization of the problem-solving activity. If the node is in an ‘open’ state (Fig. 1B, bottom), its state can be changed depending on two simultaneous conditions: (1) the node is not influenced by any of its neighboring ‘open’ nodes (each occurring with probability $1-\upbeta$), and (2) the node switches to a ‘closed’ state (with probability $\updelta$). Condition 2 reflects the fact that, with the absence of nearest-neighbor influences, each group attempts to solve its subproblem in a self-directed way. As with the reopening probability $\upbeta$, the strength of the self-directed probability $\updelta$ affects the synchronization of the problem-solving activity. Without loss of generality, we assume homogeneity with ${\upbeta }_{\mathrm{i}}=\upbeta$ and ${\updelta }_{\mathrm{i}}=\updelta$ for all nodes in the network—considered as typical average values.

**Figure 2:**
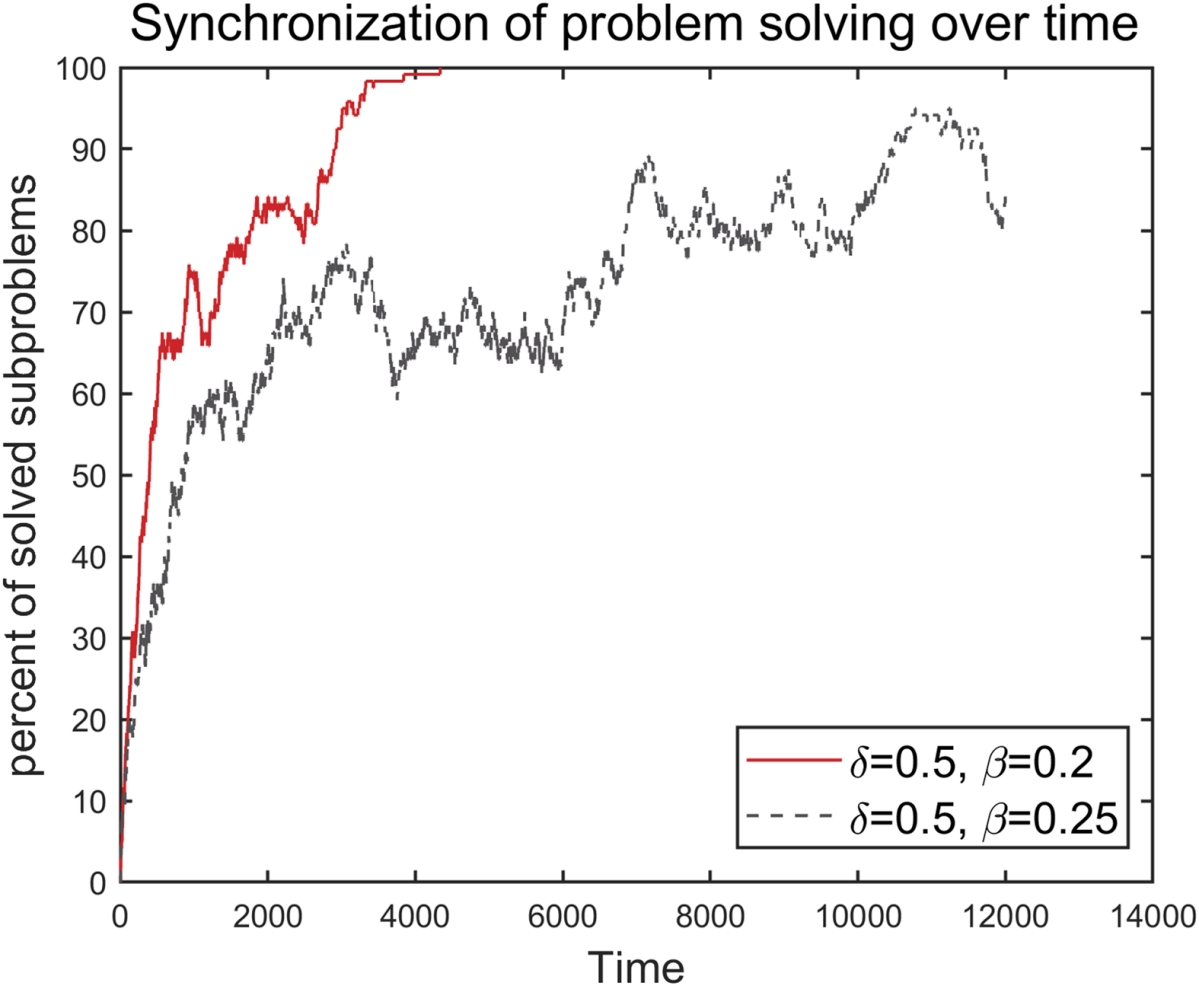
Typical simulation runs of the dynamic problem-solving model on a real-world vehicle problem-solving network with 120 nodes (subproblems) and 417 links (information flows). The graphs show the percentage of solved subproblems over time, for two simulation scenarios.

As the problem-solving activity evolves, ‘open’ subproblems are resolved, and may be reopened due to influences propagated by nearest-neighbor ‘open’ nodes. A mean-field analysis of large-scale problem-solving networks shows that—depending on $\upbeta$, $\updelta$, and the topology of the network—the process continues until either all subproblems are solved and full synchronization is achieved, or until the network settles into a quasi-equilibrium state with a non-zero fraction of ‘open’ subproblems^2^. The latter outcome reflects partial synchronization, and is an undesirable characteristic of the problem-solving activity. To illustrate this dynamical behavior, we show in Fig. 2 two typical simulation runs of the dynamic network model. The underlying network in this case is a real-world vehicle problem-solving network (see “Data” section), which includes 120 nodes (subproblems) and 417 links. We fix the self-directed probability $\updelta =0.5$, and vary the reopening probability $\upbeta$. Two different types of dynamical behavior are seen: while rapid synchronization is obtained for $\upbeta =0.2$, increasing the reopening probability to $\upbeta =0.25$ results in poor synchronization.

**Figure 3:**
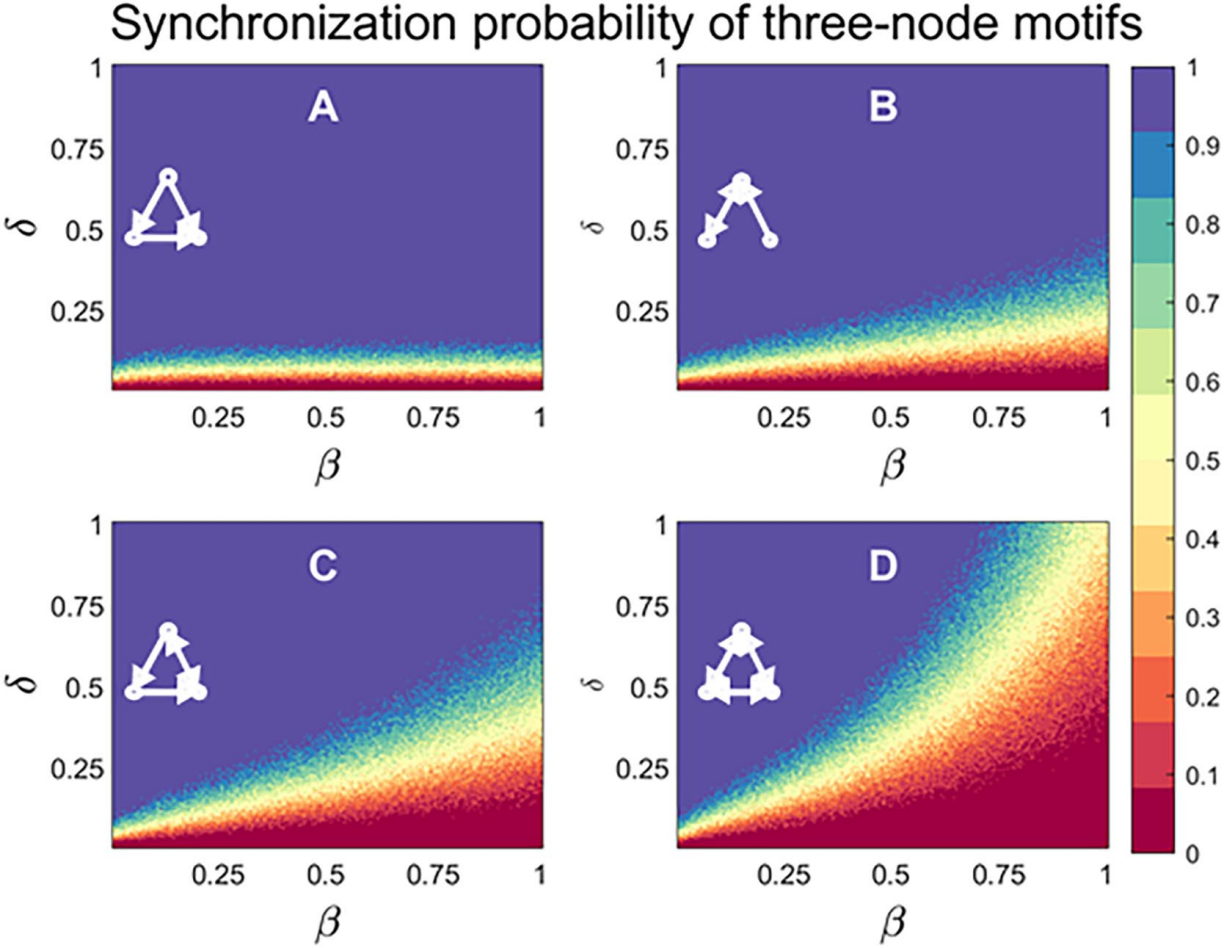
Synchronization probability of three-node subgraphs. The dynamic properties of several three-node subgraphs, with increasing number and length of feedback loops, are explored: Feedforward Loop (**A**), Mutual-In (**B**) Mutual Cascade (**C**), and Clique (**D**). The synchronization probability for a given pair of parameters $\upbeta$ and $\updelta$ is determined by generating 100 realizations of the problem-solving model, and calculating the percentage of simulations in which full synchronization (i.e. all subproblems are solved) is achieved after an arbitrarily chosen number of 120 iterations. The synchronizability metric score (SM-metric) of a subgraph is obtained by averaging over 10,000 samples of the two parameters $\upbeta$ and $\updelta$.

The dynamic analysis can also be applied at the level of a single network subgraph. For a given pair of parameters $\upbeta$ and $\updelta$, we apply the stochastic problem-solving model to a network subgraph and estimate (using Monte Carlo simulations) the probability of full synchronization after an arbitrarily chosen number of iterations. The effect of $\upbeta$ and $\updelta$, as they vary from $0$ to $1$, on the synchronization properties of several three-node network subgraphs is illustrated in Fig. 3. In general, different combinations of the two parameters $\upbeta$ and $\updelta$ result in different probabilities of subgraph’s synchronizability. For the two parameter values this is indicated by the heatmap in Fig. 3, which shows distinct dynamic properties exhibited by different three-node subgraphs. Intuitively, we see that the feedforward loop subgraph (Fig. 3A) is more likely to synchronize for a wide range of parameter values than the mutual-in subgraph (Fig. 3B), which includes a single two-node feedback loop. The mutual-in subgraph in turn is more likely to synchronize than other subgraphs that include a mixture of more complicated feedback loops (e.g. the mutual-cascade and the clique subgraphs in Fig. 3C,D). In the real world, model parameters are not constants but vary according to some distribution that depends on a variety of factors. In order to operationalize the concept of subgraph’s synchronizability, we sample a large number of parameter values $\upbeta$ and $\updelta$ from a uniform $(0, 1)$ distribution, and compute the average synchronization probability over all realizations. This results in a synchronizability metric score (SM-score), which is assigned to all possible three- or four-node subgraphs, and then compared with subgraph frequency in real problem-solving networks.

## Data

We compiled well-characterized data from the literature, quantifying relationships between subproblems tasks in diverse problem-solving environments and geographical locations. The original data is part of a commonly used method (Dependency Structure Matrix, DSM), which uses a matrix representation of a directed graph to graphically depict information dependencies between the elements of a complex system^25–29^. Mapping the interdependencies among subproblem tasks in these data was conducted primarily using structured interviews with experienced individuals involved in the problem-solving activity. The problem-solving network data considered here include: vehicle development (‘Veh1’, see Supplementary Data S1 online) with 120 nodes and 417 directed links^2^; real estate development (‘Red’, see Supplementary Data S2 online) with 91 nodes and 1148 directed links^63^; strategy and knowledge development (‘Knwl’, see Supplementary Data S3 online) with 62 nodes and 285 directed links^64^; microprocessor development (‘Mip’, see Supplementary Data S4 online) with 60 nodes and 301 directed links^65^; bioscience facility development (‘Bio’, see Supplementary Data S5 online) with 53 nodes and 230 directed links^66^; vehicle development (‘Veh2’, see Supplementary Data S6 online) with 44 nodes and 249 directed links^67^; and equipment development (‘Equip’, see Supplementary Data S7 online) with 43 nodes and 120 directed links^68^.

## Results

**Figure 4:**
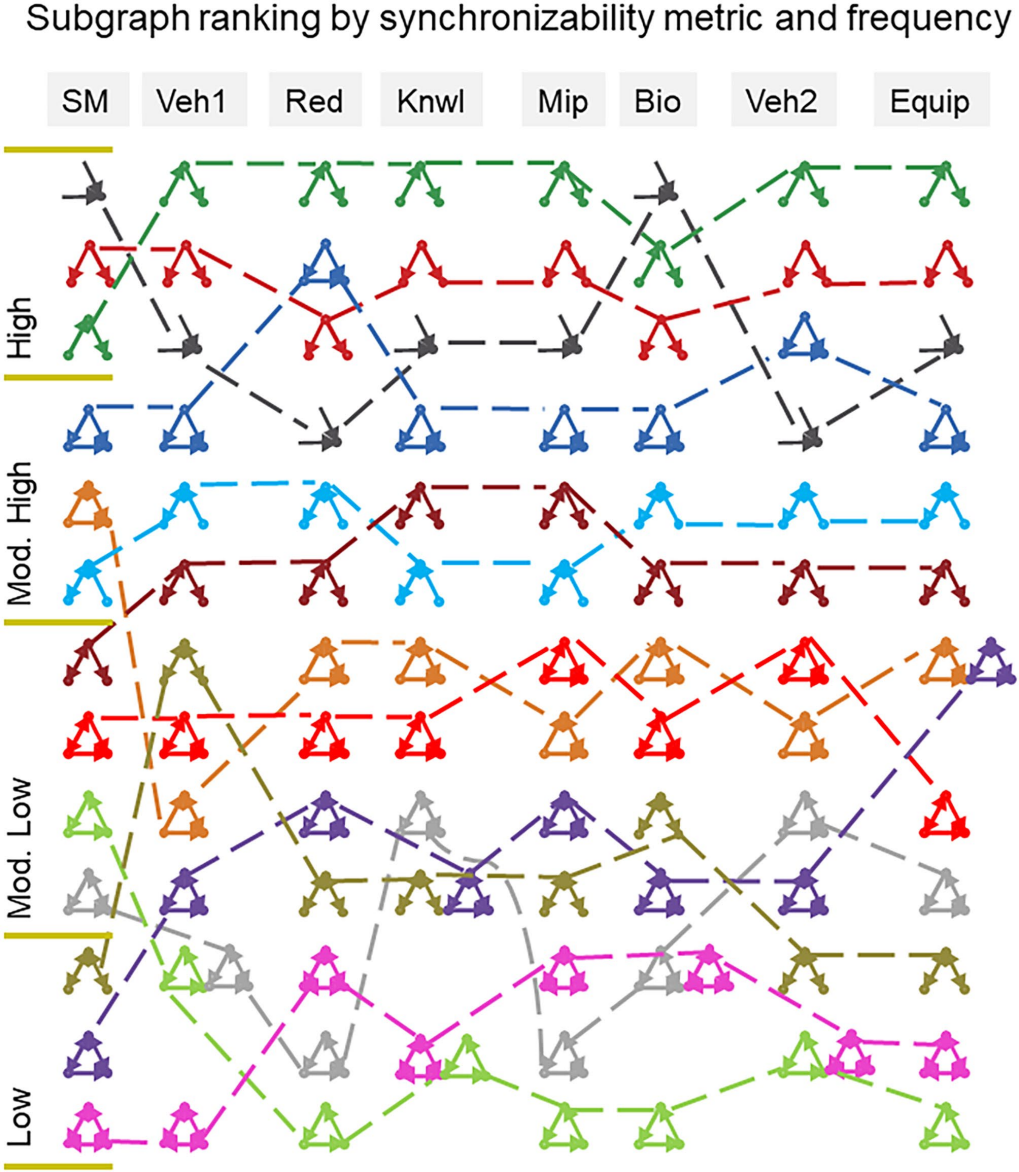
Ranking of all topologically distinct 13 three-node subgraphs according to their SM scores and occurrence in real problem-solving networks. The three-node subgraphs are ranked from largest to smallest SM scores (first column, top to bottom). We use the quartiles of the SM scores to divide the subgraphs into four natural SM classes (low SM score, moderately-low SM score, moderately-high SM score, and high SM score). The ranking of subgraphs, from largest to smallest frequencies (top to bottom), are shown in columns 2–8 where each column represents a particular real network (networks are ordered from larger to smaller). A monotonic association between synchronizability and subgraph frequency as well as between subgraph frequency in different problem-solving networks is suggested by the graphical representation. More rigorous nonparametric statistical analyses (Spearman’s correlation and Kruskal–Wallis tests) are used in the text to substantiate this suggestion.

We calculate the SM scores for all topologically distinct 13 three-node and 199 four-node directed subgraphs. We further enumerate the frequency of all three- and four-node subgraphs in each of the seven real problem-solving networks, where the frequency of a subgraph in a particular network is the number of different matches of this subgraph. The frequency of subgraphs is then compared with their assigned SM scores. To get a sense of the relative order between the various subgraphs, we rank the three-node subgraphs by their assigned SM scores (largest to smallest) as well as by their frequencies (largest to smallest) in the real networks (Fig. 4). As Fig. 4 shows, a strong relationship is suggested between synchronizability and subgraph frequency. In general, subgraphs that are more synchronizable tend to be more abundant in the real networks. Of no less importance is the fact that the ranking of subgraph frequency is quite consistent across the diverse problem-solving networks, suggesting that the non-random nature of problem-solving networks is closely linked to the synchronizability of network subgraphs.

**Figure 5:**
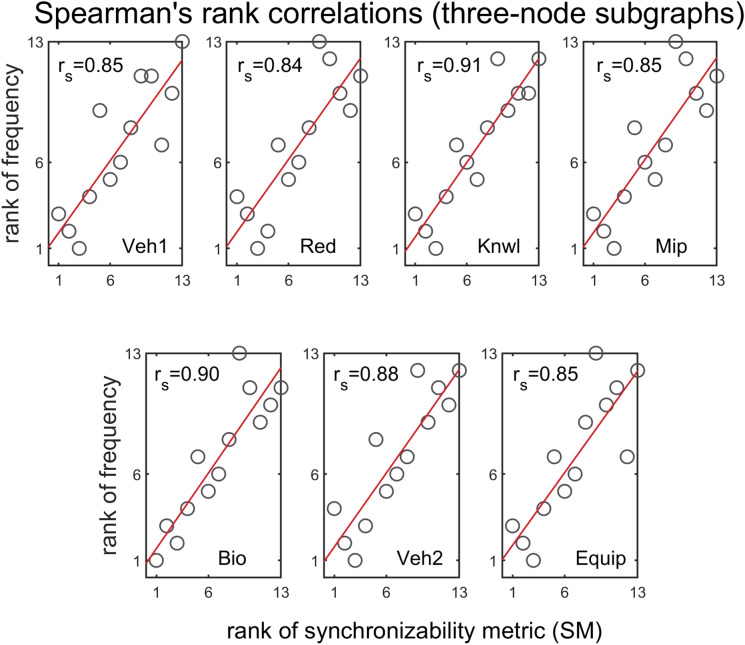
Spearman's rank correlations between three-node subgraph SM score and three-node subgraph frequency, for all real problem-solving networks. The panels show scatter plots of ranks of subgraph frequencies (ranking from high to low) versus ranks of SM scores (ranking from high to low), for each of the real networks.

**Figure 6:**
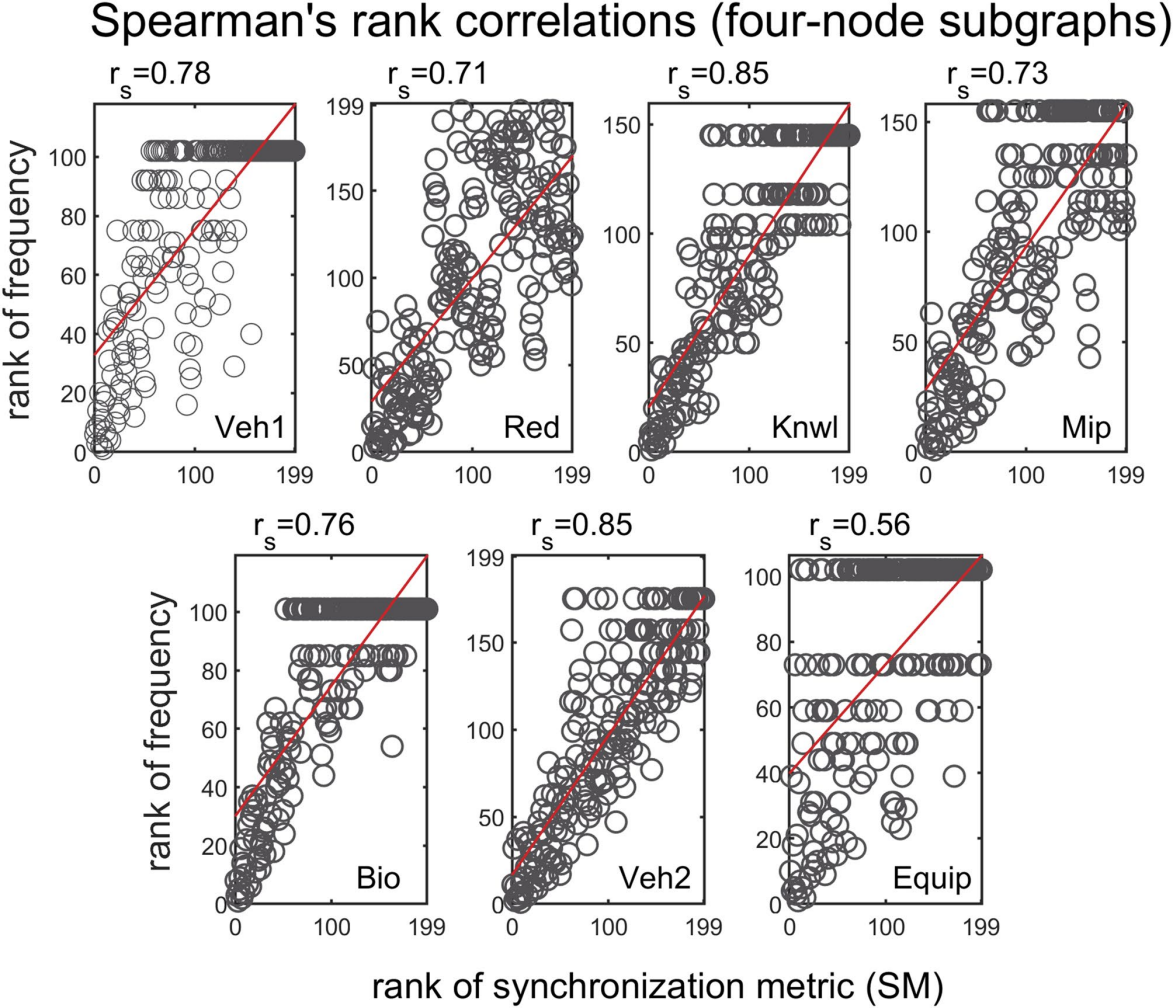
Spearman's rank correlations between four-node subgraph SM score and four-node subgraph frequency, for all real problem-solving networks. The panels are as in Fig. 5.

We apply several statistical tests to further substantiate the above observations. A series of Spearman rank correlations were conducted in order to determine if there were any relationships between SM score and subgraph frequency, in different problem-solving networks. Spearman’s rank correlation measures the strength and direction of monotonic association between two variables, and is determined by calculating Pearson’s correlation on the ranked values of the data. Figure 5 shows the Spearman’s correlation coefficients for three-node subgraphs. A two-tailed test of significance indicates the there is a strong monotonic relationship between SM score and subgraph frequency ($0.84{\le r}_{s}(13)\le 0.91$, $\mathrm{p}<0.001$). On average, the higher the SM score of a subgraph, the more abundant the subgraph in the real network. The results are extended to four-node subgraphs as shown in Fig. 6. The 199 four-node subgraphs offer a fuller description of patterns of local interconnections than the three-node subgraphs, and can help refine the association between synchronizability and subgraph frequency. Results of the Spearman correlations indicate that there is a strong monotonic association between SM score and subgraph frequency (the vast majority of ${\mathrm{r}}_{\mathrm{s}}(199)$ are between $0.71$ and $0.85$, $\mathrm{p}<0.001$). Surprisingly, even with the increased variability introduced by four-node subgraphs, the general trend is the same as in the three-node analysis.

**Figure 7:**
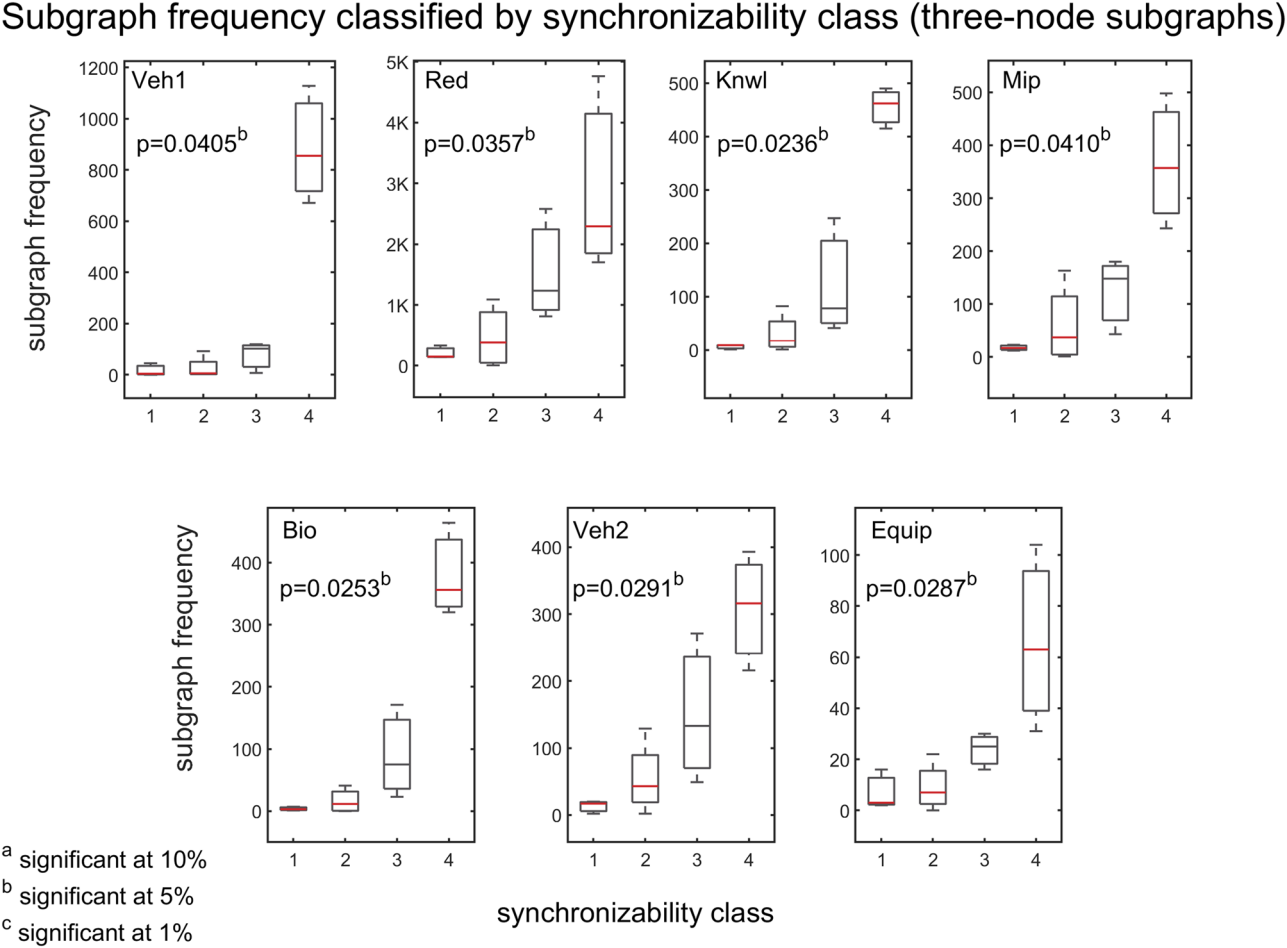
Box plots of three-node subgraph frequencies contrasted by subgraph synchronizability class. Synchronizability classes 1, 2, 3, and 4 correspond to low SM score, moderately-low SM score, moderately-high SM score, and high SM score, respectively. The box plot is a five-number summary of the empirical distribution^69–73^. The outer edges of the box represent the first quartile $\mathrm{Q}1$ (the 25th percentile), and the third quartile $\mathrm{Q}3$ (the 75th percentile). The middle red line of the box indicates the median (or the 50th percentile). The length of the box, $\mathrm{Q}3-\mathrm{Q}1$, is the interquartile range (IQR), which measures the spread in the data. The dashed line (“upper whisker”) that extends from $\mathrm{Q}3$ is the smallest between the maximum value of the sample and $\mathrm{Q}3+1.5\times \mathrm{IQR}$, and the dashed line (“lower whisker”) that extends from $\mathrm{Q}1$ is the largest between the minimum value of the sample and $\mathrm{Q}1-1.5\times \mathrm{IQR}$. Observations that are farther than $1.5\times \mathrm{IQR}$ from the top or bottom of the box indicate outliers, and are shown as red + signs. The p‐value of the Kruskal–Wallis tests is less than 0.05, indicating a statistically significant difference in subgraph frequency between the different synchronizability classes. Each panel corresponds to a particular problem-solving network.

**Figure 8:**
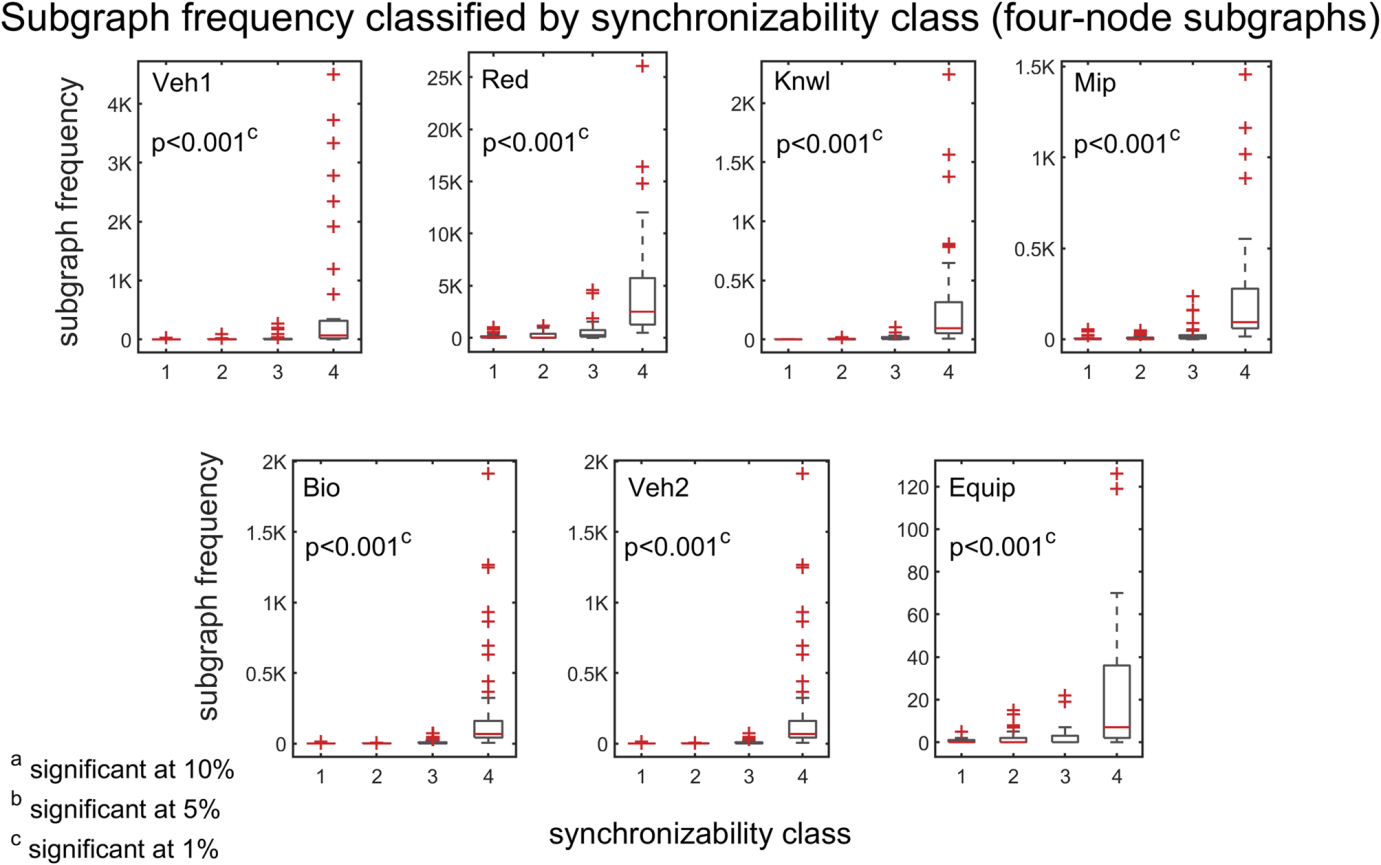
Box plots of four-node subgraph frequencies contrasted by subgraph synchronizability class. The box plots and panels are defined as in Fig. 7.

To further explore the association between synchronizability and subgraph frequency, we use the quartiles of the SM scores to divide the subgraphs into four natural synchronizability classes (high SM score, moderately-high SM score, moderately-low SM score, and low SM score). Figure 7 presents box plots of the frequencies of three-node subgraphs (grouped by the different synchronizability classes), for all of the real problem-solving networks. Figure 8 shows the results for four-node subgraphs. A box plot (also called a box-and-whisker plot) is a standard graphical tool (see caption of Fig. 7 for details) used in statistics^69^ and other quantitative sciences^70–73^ to visualize summary statistics for sample data^69^, compare groups of data^70–72^, or identify extreme events^73^. On average, the box plots indicate that the higher the synchronizability class of subgraphs, the higher the frequencies of the subgraphs in the real network. A Kruskal–Wallis test^70^—a nonparametric alternative to the one-way ANOVA—shows that there is a statistically significant difference in subgraph frequency between the different synchronizability classes ($\mathrm{p}\le 0.05$ for three-node subgraphs, and $\mathrm{p}<0.001$ for four-node subgraphs).

Although the above results are revealing, there might be confounding and selection biases acting on the network’s subgraphs, which could introduce spurious relationships between subgraph synchronizability and subgraph frequency. To avoid any such selective biases, we extend the previous analysis by controlling for possible confounding factors. First, the unobserved mechanism that drives the organization of the problem-solving network might be a confounding factor. Therefore, we employ a random null model and focus on the statistical significance of subgraphs rather than looking at their counts in the network. The statistical significance of a subgraph is determined by comparing its frequency in the real network relative to its mean frequency in an ensemble of a large number of randomly generated networks^46–48,52^. Statistically significant subgraphs (also called network motifs) are defined as subgraphs that occur in a real network much more often than in random networks. Using a random null model, which is free of any type of selective bias, will enable to adequately account and control for the non-random nature of problem-solving networks, and test the hypothesis that subgraphs with higher-synchronizability are more overrepresented—when compared to randomized networks—in real problem-solving networks.

To generate the ensemble of randomized networks, we employ the simplest Erdös-Rényi (ER) random graph model^32^, which was used before to detect network motifs^48,49^. To make a meaningful comparison, each simulated random network in the ensemble is constrained to have the same number of nodes and directed links as in the corresponding real network. The choice of the ER random graph model is motivated by the fact that it is devoid of any organizing principles^48,49^. This reduces the risk of confounding by unmeasured factors, and makes the association between subgraph abundance and subgraph dynamics clearer. The statistical significance of each (three- or four-node) subgraph is measured by calculating the Z-score, which is defined as the difference between the subgraph frequency in the real network and the mean frequency in a large ensemble of randomly generated ER networks, divided by the standard deviation of the frequency values for the randomized networks^46–48,52^. The Z-scores of three-node subgraphs was used in ^47^ to calculate the significance profile of a directed network, obtained by normalizing the vector of Z-scores. These methods were found to be useful in clustering networks into distinct families based on the correlations between their significance profiles^47^. Since the most abundant subgraphs in sparse networks tend to have fewer edges than less abundant subgraphs^48,49^, it is necessary in subsequent analysis to also control and eliminate the influence of edge number on subgraph significance, when studying the relationship between the significance of three- and four-node subgraphs and their dynamic properties. This is achieved, as suggested in^49^, by dividing the subgraphs into density classes, each of which containing subgraphs with the same number of directed links.

**Figure 9:**
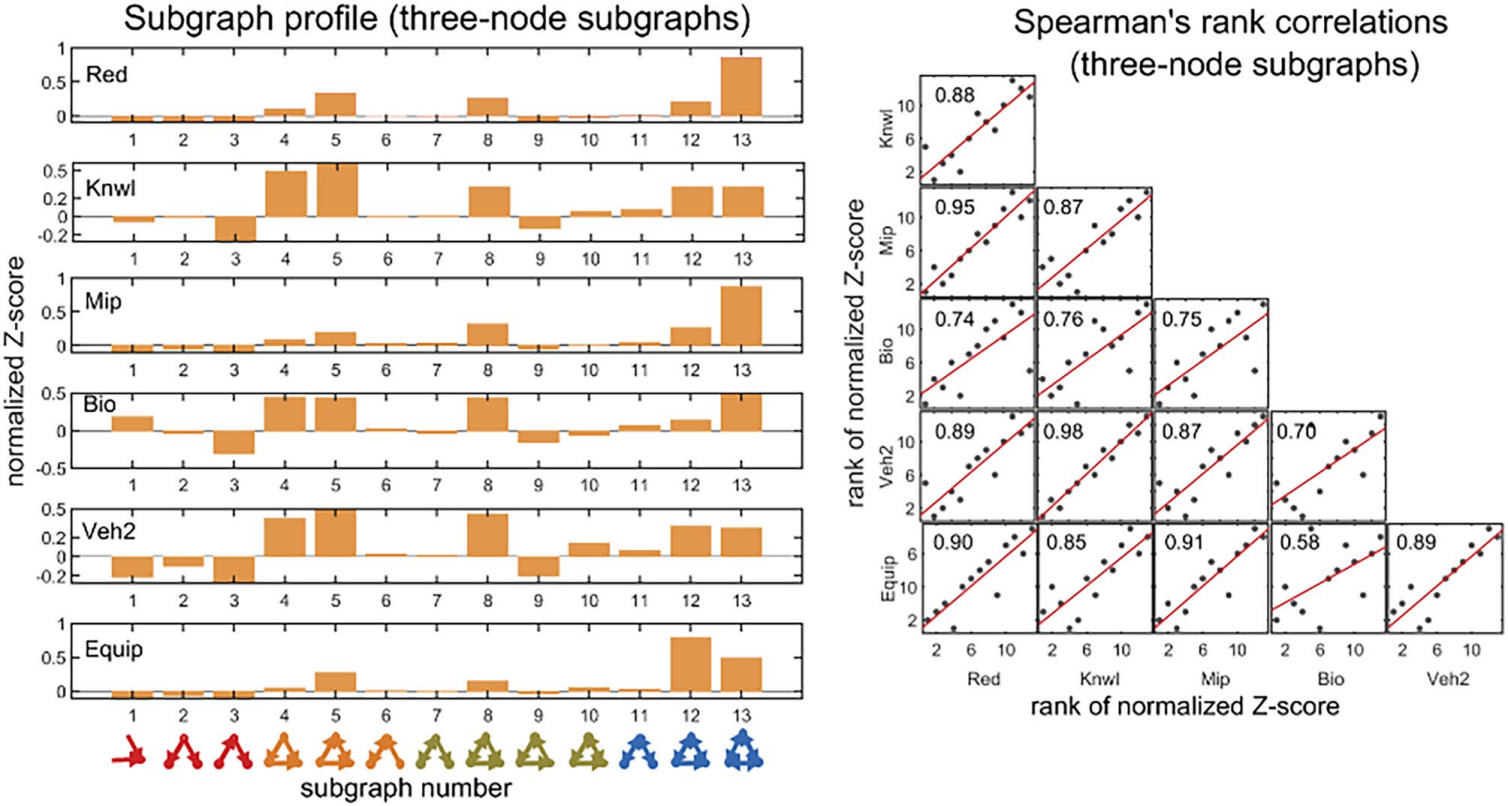
Spearman’s rank correlation matrix (right panel) of the three-node significance profiles (left panel) for the directed problem-solving networks (‘Red’ through ‘Equip’; for comparison, ‘Veh1’ was excluded due to an undefined Z-score of subgraph 13). (Left panel) The three-node significance profiles for the problem-solving networks. The significance profile shows the normalized Z-score for each of the 13 connected subgraphs. (Bottom of left panel) The 13 connected subgraphs are ordered from left to right by their SM scores, and each is colored according to its synchronizability class (red, yellow, green, and blue corresponding to high, moderately-high, moderately-low, and low SM scores, respectively). The normalized Z-score of all subgraphs is determined by comparison to 500 randomized ER networks. (Right panel) Spearman’s rank correlations among pairs of significance profiles. The panels show scatter plots of ranks of subgraph normalized Z-scores in one network (ranking from high to low) versus ranks of subgraph normalized Z-scores in another network (ranking from high to low).

Before presenting the main findings, it is instructive to examine the significance profiles of the 13 possible connected subgraphs for the real problem-solving networks. Figure 9 (left panel) shows the significance profiles of the 13 possible three-node subgraphs for the problem-solving networks. Results of Spearman correlations (Fig. 9, right panel) of the significance profiles indicate a strong monotonic relationship between subgraph abundance in different unrelated problem-solving networks (the vast majority of ${\mathrm{r}}_{\mathrm{s}}(13)$ are between $0.7$ and $0.98$, $\mathrm{p}<0.001$), furthering the hypothesis that subgraph abundance is driven by dynamic properties of local network structures. Subgraphs 4, 5, 8, 12 and 13 have the highest normalized Z-scores, and subgraphs 3 and 9 the lowest. The feedforward loop (subgraph 4) is composed of a problem-solving activity that sends information to another activity, and both send information to a third activity. The regulated and regulating mutual subgraphs (subgraphs 5 and 8, respectively) show mutual information feedback between two activities that send to or receive information from a third activity. The semi-clique (subgraph 12) and clique (subgraph 13) display three activities that repeatedly interact with each other, either directly or via transitive interactions. The cascade (subgraph 3) and the feedback loop (subgraph 9) are rare. What could explain these patterns?

The bar graphs (Fig. 9, left panel) show the normalized Z-scores of the 13 subgraphs, which are ordered from left to right by their SM scores. Subgraphs 1, 2, and 3 represent a density class with 2 edges; subgraphs 4, 6, 7, and 9 represent a density class with 3 edges; subgraphs 5, 8, 10, and 11 represent a density class with 4 edges; and subgraphs 12 and 13 represent density classes with 5 and 6 edges, respectively. Remarkably, the comparison of the significance of subgraphs within a particular density class reveals a general trend of significant overrepresentation of subgraphs with higher synchronizability, and weak overrepresentation—and sometimes underrepresentation—of subgraphs with lower synchronizability. Subgraphs 1 and 2 consistently have higher normalized Z-scores than subgraph 3. In almost all cases, the order of normalized Z-scores among pairs of 3-edge subgraphs (4, 6, 7, and 9) is consistent with their SM score ranking. Subgraph 4, which has the highest SM score, is significantly overrepresented in all networks relative to all other 3-edge subgraphs; and subgraph 9, which has the lowest SM score, is underrepresented in all networks relative to other 3-edge subgraphs. In almost all cases, the order of normalized Z-scores among pairs of 4-edge subgraphs (5, 8, 10, and 11) is consistent with their SM score ranking. Subgraphs 5 and 8, with moderate SM scores, are significantly more overrepresented than subgraphs 10 and 11, which have lower SM scores.

**Figure 10:**
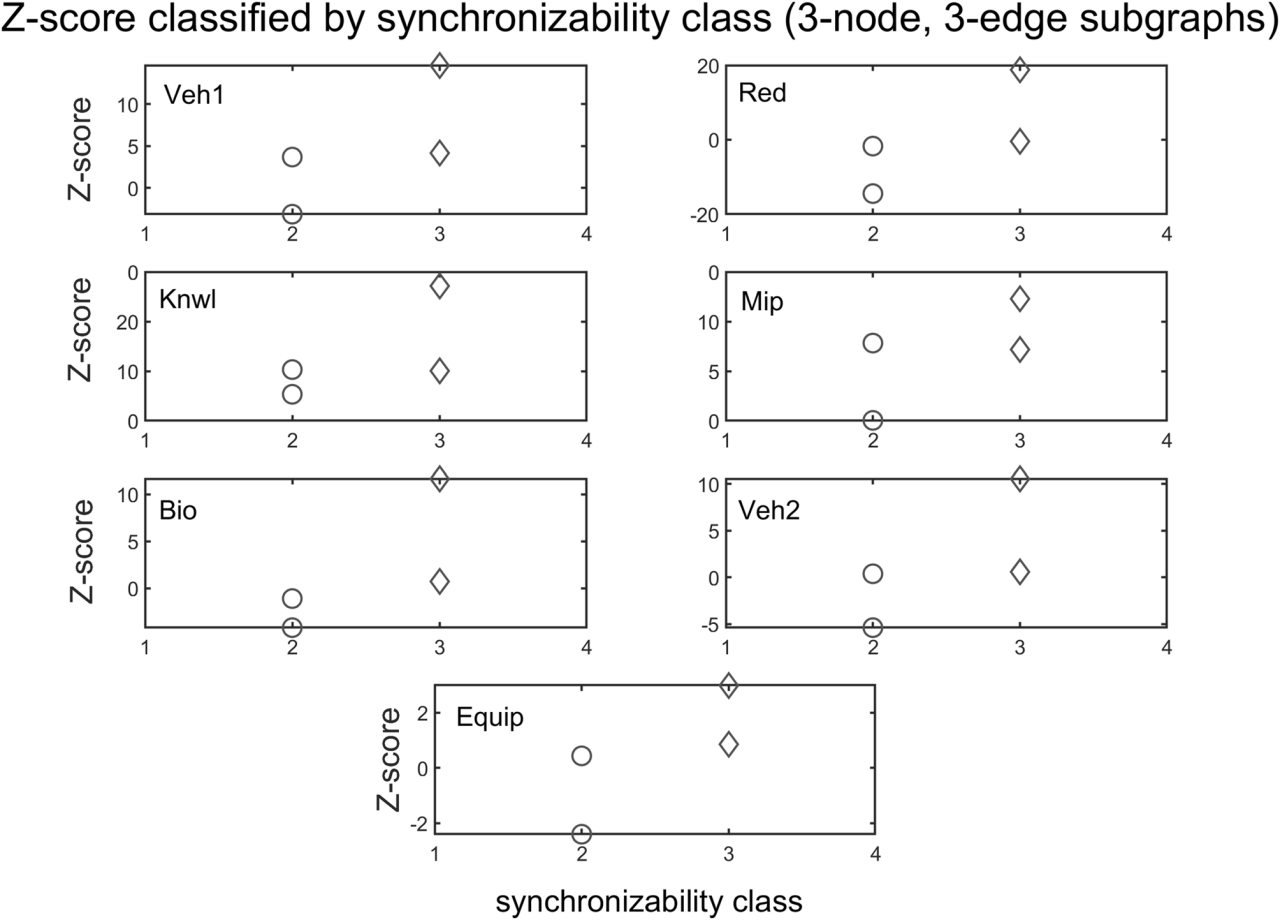
Z-score classified by synchronizability class, for three-node subgraphs in the 3-edge density class. The panels show scatter plots of subgraph SM class (SM classes 1, 2, 3, and 4 correspond to low, moderately-low, moderately-high, and high SM scores, respectively) versus subgraph Z-score, for each of the real networks.

**Figure 11:**
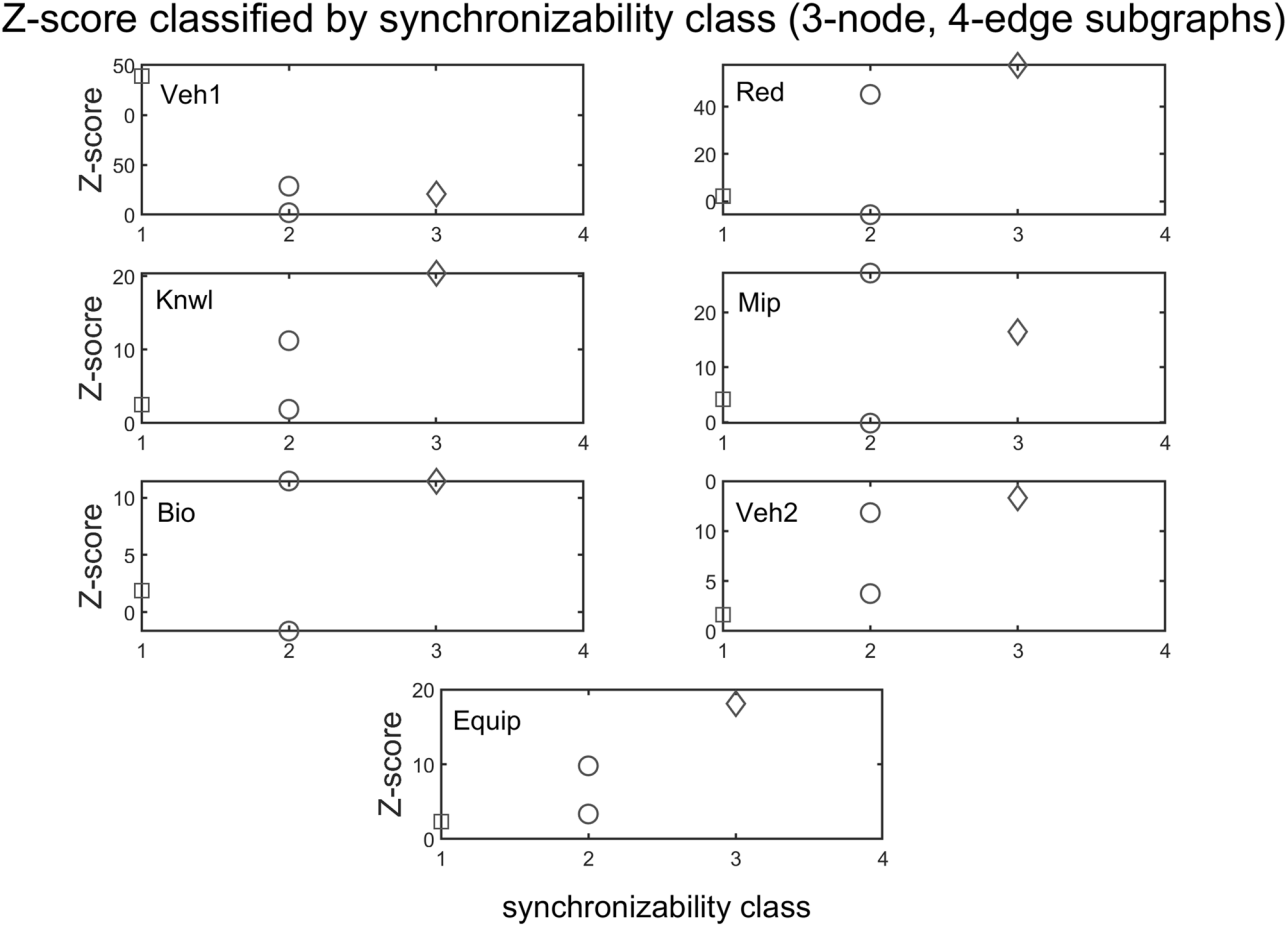
Z-score classified by synchronizability class for three-node subgraphs in the 4-edge density class. The panels show scatter plots of subgraph SM class (SM classes are numbered as in Fig. 10) versus subgraph Z-score, for each of the real networks.

**Figure 12:**
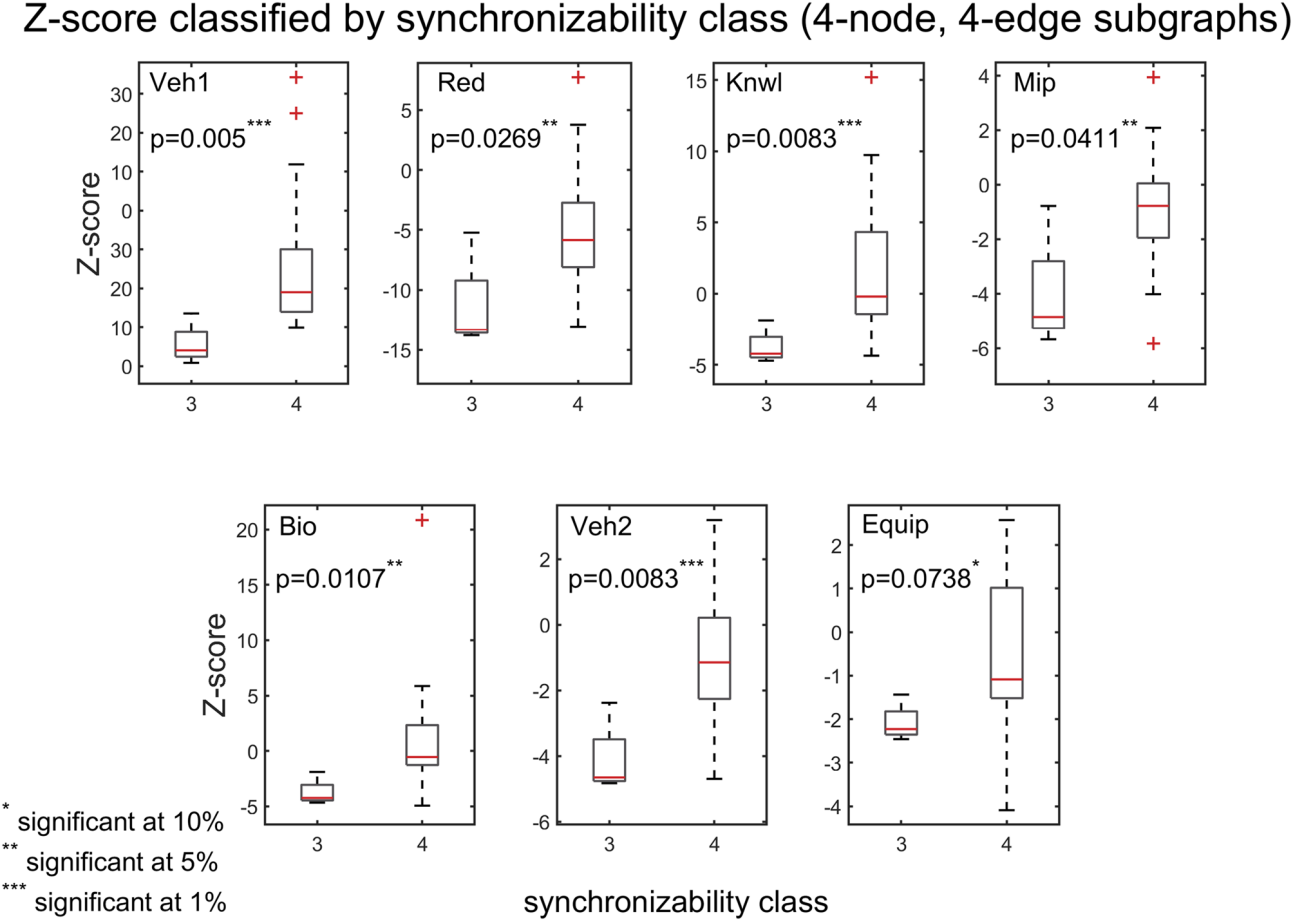
Box plots of Z-scores, for four-node, 4-edge subgraphs, contrasted by subgraph SM class (SM classes are numbered as in Fig. 10). In most cases, the p‐value of the Kruskal–Wallis tests is less than 0.05, indicating a statistically significant difference in subgraph abundance (relative to a random null model) between the different synchronizability classes. Each panel corresponds to a particular problem-solving network. The box plots are defined as in Fig. 7.

**Figure 13:**
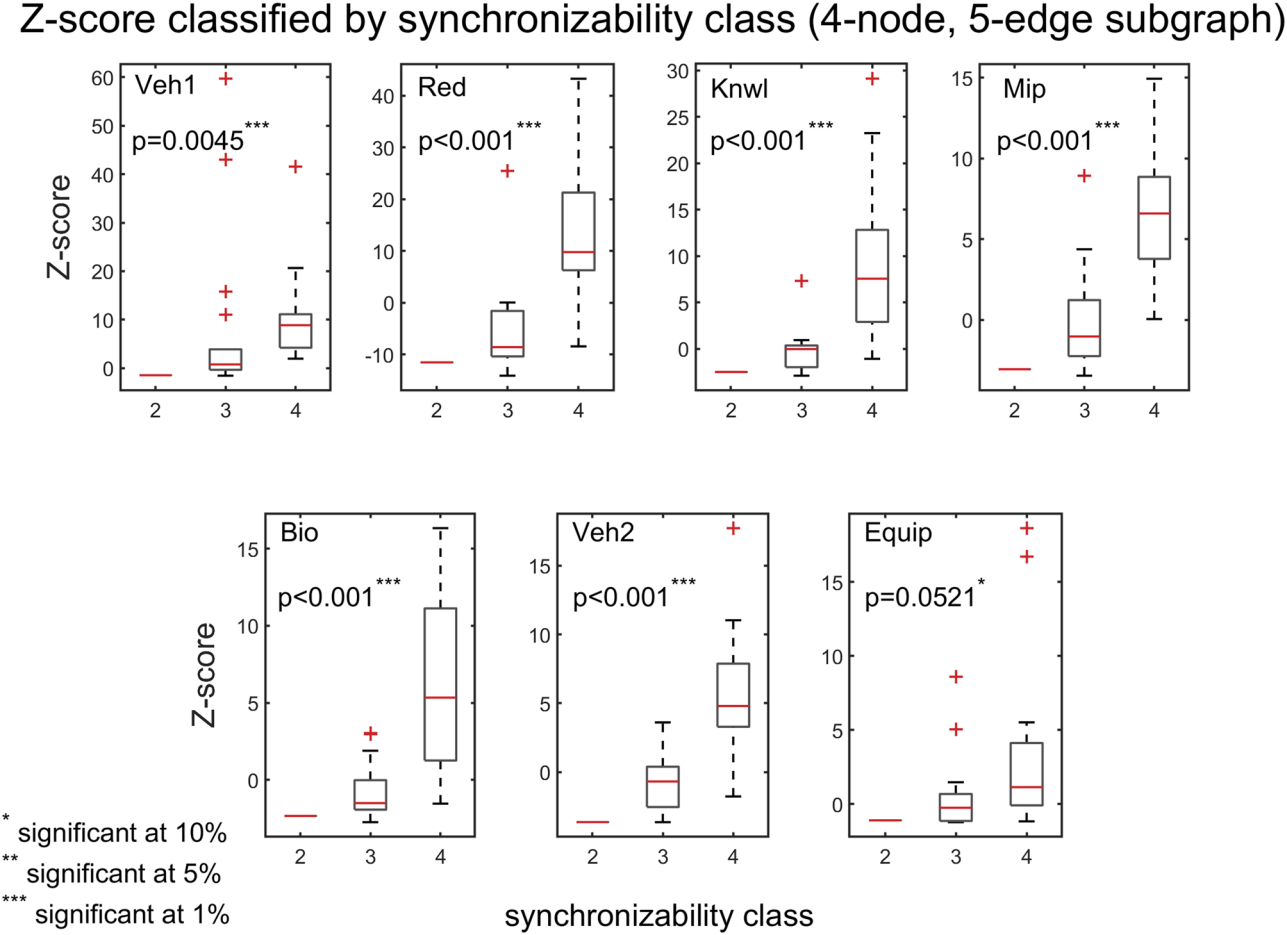
Box plots of Z-scores, for four-node, 5-edge subgraphs, contrasted by subgraph synchronizability class. Details and panels are as in Fig. 12. In most cases, the p‐value of the Kruskal–Wallis tests is less than 0.05, indicating a statistically significant difference in subgraph abundance (relative to a random null model) between the different synchronizability classes. The box plots are defined as in Fig. 7.

**Figure 14:**
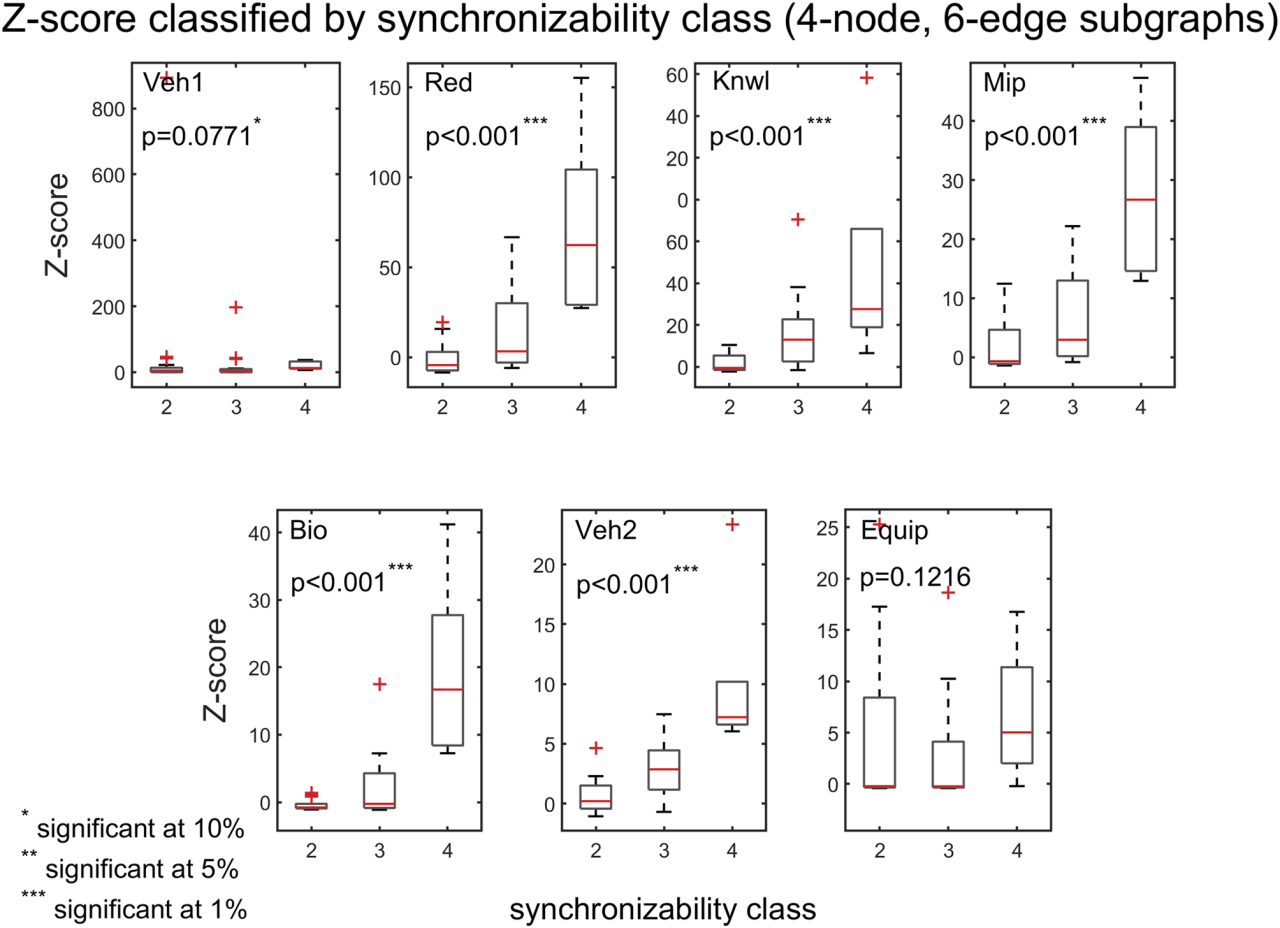
Box plots of Z-scores, for four-node, 6-edge subgraphs, contrasted by subgraph synchronizability class. Details and panels are as in Fig. 12. In most cases, the p‐value of the Kruskal–Wallis tests is less than 0.05, indicating a statistically significant difference in subgraph abundance (relative to a random null model) between the different synchronizability classes. The box plots are defined as in Fig. 7.

The interplay between subgraph abundance and synchronizability can also be seen by comparing the SM classes of subgraphs, within a particular density group, with their Z-scores. The 2-edge subgraphs (1, 2, and 3) belong to the high SM class, and both the 3-edge subgraphs (4, 6, 7, and 9) and 4-edge subgraphs (5, 8, 10, and 11) belong to multiple SM classes (Fig. 9, bottom left). Figures 10 and 11 show scatter plots of Z-score versus SM class for 3-edge and 4-edge subgraphs, respectively. We see that in almost all cases subgraphs of higher SM classes are more overrepresented in real problem-solving networks than subgraphs of lower SM classes. The complexity and variety of the 199 four-node directed subgraphs enables a more comprehensive statistical analysis of the relationship between subgraph abundance and subgraph synchronizability. A series of Kruskal–Wallis tests, one for each subgraph density class, are conducted to examine this relationship for four-node subgraphs. Figures 12, 13, 14 show that—in each of the 4-edge, 5-edge, and 6-edge density classes—there is a statistically significant difference in subgraph Z-score between the different synchronizability classes (p ≤ 0.05 for most cases). The comparison of the dynamic properties of subgraphs to their Z-scores reveals that—compared to a random null model—subgraphs with higher synchronizability are more enhanced in the real networks, while low- synchronizable subgraphs are more suppressed. This suggests that the dynamic properties of network subgraphs have an influence on their abundance in the network and correspondingly the overall organization of the problem-solving network.

Although the Z-score is a commonly used measure for detecting statistically significant subgraphs^46–48,51^, it does not provide full information on the relative importance of subgraphs. The relative importance of a subgraph can also be understood in terms of the relative difference between the subgraph frequency in the target network and the expected frequency in the random networks. One way to define the relative difference of two numbers is to take their difference divided by some function of the two numbers^74^. Following^47,74^, we define the relative difference (RD-score) of each (three- or four-node) subgraph as the difference between the subgraph frequency in the real network and the mean frequency in ER networks, divided by the sum of these two frequencies. In order to compare networks of different sizes, the vector of subgraph RD-scores can be normalized, obtaining the RD profile of the directed network^47^. The Z and RD scores do not necessarily overlap^47^—a subgraph can be detected as statistically significant (high Z-score) due to a narrow distribution of subgraph incidence in the randomized networks, but still have a slight difference between its abundance in the real network relative to random networks (small RD-score).

**Figure 15:**
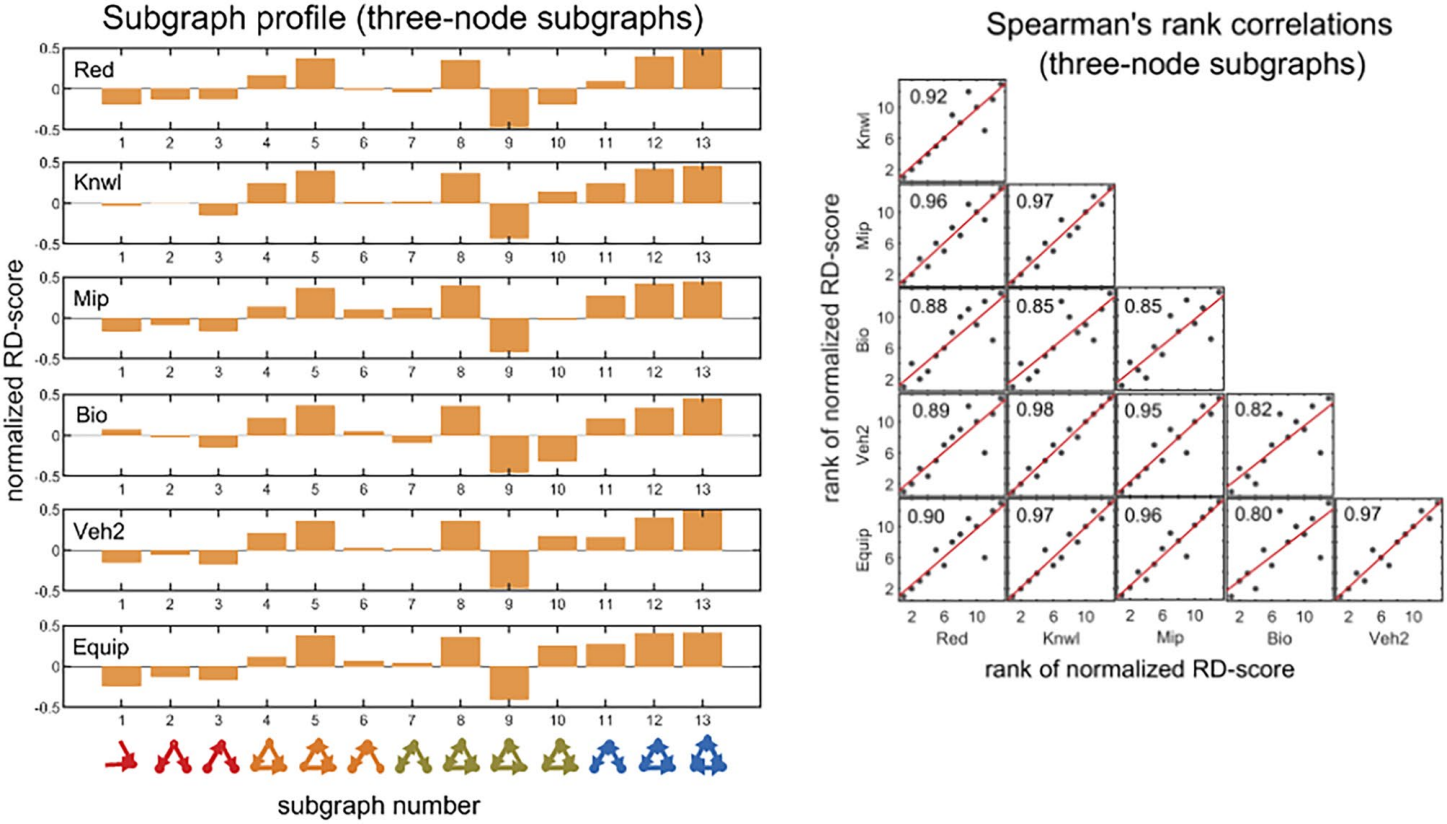
Spearman’s rank correlation matrix (right panel) of the three-node RD profiles (left panel) for the directed problem-solving networks (‘Red’ through ‘Equip’; for comparison, ‘Veh1′ was excluded due to an undefined RD-score of subgraph 13). (Left panel) The three-node RD profiles for the problem-solving networks. The RD profile shows the normalized RD-score for each of the 13 connected subgraphs. (Bottom of left panel) The 13 connected subgraphs are ordered from left to right by their SM scores, and each is colored as described in Fig. 9. The normalized RD-score of all subgraphs is determined by comparison to 500 randomized ER networks. (Right panel) Spearman’s rank correlations among pairs of RD profiles. The panels show scatter plots of ranks of subgraph normalized RD-scores in one network (ranking from high to low) versus ranks of subgraph normalized RD-scores in another network (ranking from high to low).

**Figure 16:**
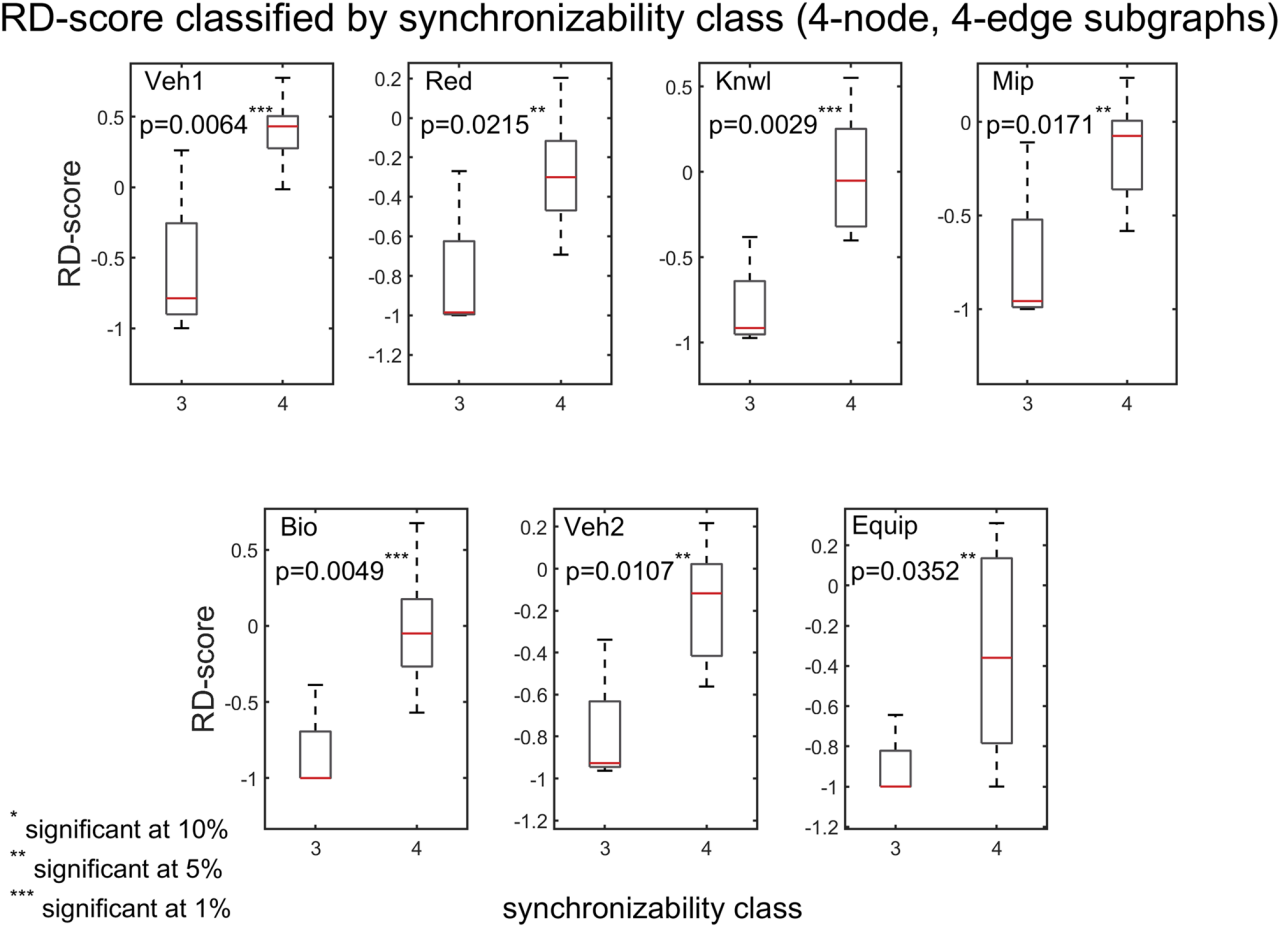
Box plots of RD-scores, for four-node, 4-edge subgraphs, contrasted by subgraph SM class (SM classes are numbered as in Fig. 10). In all cases, the p‐value of the Kruskal–Wallis tests is less than 0.05, indicating a statistically significant difference in subgraph RD-score between the different synchronizability classes. Each panel corresponds to a particular problem-solving network. The box plots are defined as in Fig. 7.

**Figure 17:**
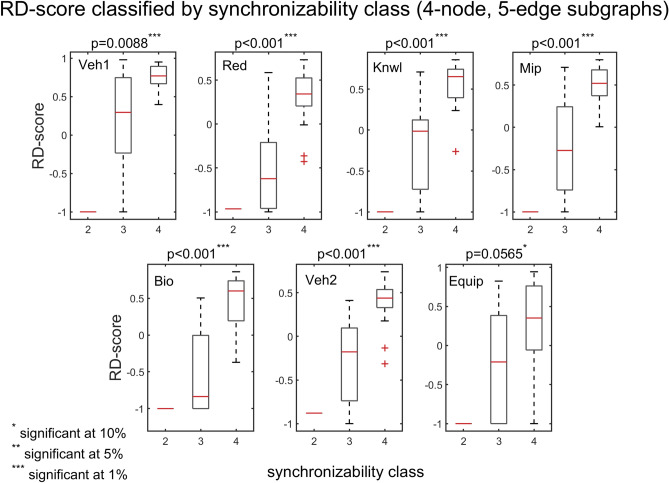
Box plots of RD-scores, for four-node, 5-edge subgraphs, contrasted by subgraph synchronizability class. Details and panels are as in Fig. 16. In most cases, the p‐value of the Kruskal–Wallis tests is less than 0.05, indicating a statistically significant difference in subgraph RD-score between the different synchronizability classes. The box plots are defined as in Fig. 7.

**Figure 18:**
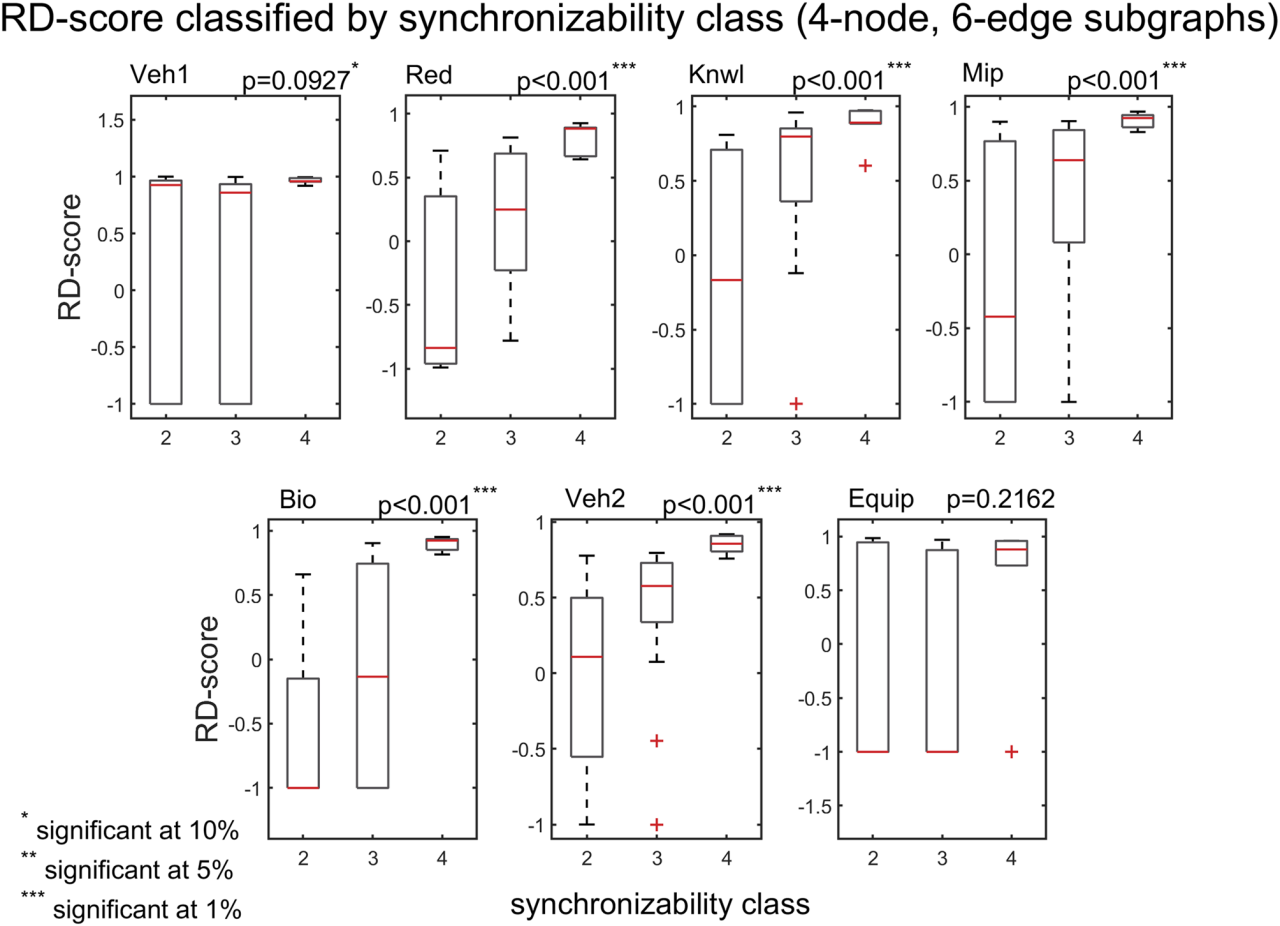
Box plots of RD-scores, for four-node, 6-edge subgraphs, contrasted by subgraph synchronizability class. Details and panels are as in Fig. 16. In most cases, the p‐value of the Kruskal–Wallis tests is less than 0.05, indicating a statistically significant difference in subgraph RD-score between the different synchronizability classes. The box plots are defined as in Fig. 7.

We compare the problem-solving networks based on the RD profiles of the 13 possible three-node subgraphs (Fig. 15, left panel). Results of Spearman correlations (Fig. 15, right panel) indicate a very strong monotonic relationship between subgraph RD-scores in different unrelated problem-solving networks (${\mathrm{r}}_{\mathrm{s}}(13)$ are between $0.8$ and $0.98$, $\mathrm{p}<0.001$), suggesting (as also implied by Fig. 9) that the networks have similar key subgraphs that were developed to have similar dynamic properties. Subgraphs 4, 5, 8, 12 and 13 have the highest normalized RD-scores, and subgraphs 3 and 9 the lowest, which is consistent with the results obtained for the Z-score. The bar graphs (Fig. 15, left panel) show the normalized RD-scores of the 13 subgraphs, which are ordered from left to right by their SM scores. Remarkably, and consistent with the results for the Z-score, the comparison of the RD-scores of subgraphs within a particular density class (i.e., 3-edge and 4-edge) reveals a general trend of large subgraph RD-scores having high SM classes, and small subgraph RD scores—and sometimes negative RD-scores—having low SM classes. This trend is further substantiated when analyzing the 199 four-node subgraphs. Figures 16, 17, 18 show that—in each of the 4-edge, 5-edge, and 6-edge density classes—there is a statistically significant difference in subgraph RD-score between the different synchronizability classes (Kruskal–Wallis, p ≤ 0.05 for most cases). The excellent correlation between subgraph synchronizability and both subgraph abundance (Z-score) and relative difference (RD-score) indicates that subgraphs with higher synchronizability are both more statistically significant and occur in real networks at numbers that are significantly larger than those in randomized networks. In other words, highly-synchronizable subgraphs are both significant and important in real problem-solving networks. This suggests, once again, that the topology of problem-solving networks is deeply related to the dynamic properties of regular patterns of local interconnections, which constitute the basic building blocks of the networks.

## Discussion

In this paper, we study the relationship between the dynamic properties of three- and four-node subgraphs and their frequency in directed problem-solving networks. The dynamic behavior of a subgraph is characterized in terms of its synchronizability—measured by the probability of rapidly converging to a problem-solving equilibrium. We give an evidence that highly synchronizable subgraphs are more overrepresented and critical in real problem-solving networks.

What is the origin of the correlation revealed between synchronizability and subgraph prevalence? Although it is difficult to fully disentangle the causative relation between the dynamical properties of subgraphs and their abundance, the results in this paper suggest that both global constraints and properties of local subgraphs influence the occurrence of subgraphs in a given problem-solving network. Collaborative problem-solving activity is organized into a nested hierarchical structure with semi-independent activities at different scales performing particular tasks that contribute to the overall performance of the network. In such a nested hierarchy, subgraphs—patterns of local collaborative activities—do not exist in isolation but are embedded in a heavily interconnected network of “subgraphs within subgraphs”, which are connected to each other via patterns of local ties that are themselves subgraphs at higher scales^30^. Moreover, as pointed out in^75,76^, real complex network connections are often highly fluid; even when there exists an underlying fixed topological structure, connections between nodes (and so subgraphs) can adaptively become active or inactive over time. In complex problem-solving networks, we might expect the dynamics of lower-level subgraphs to change faster (i.e., on a shorter time scale) than higher-level configurations. The nested hierarchical organization along with the separation of time scales exerts a powerful effect on the synchronization and convergence of the problem-solving activity. In the short run, lower-level subgraphs will tend to rapidly synchronize to an approximate equilibrium state nearly independently of others. These accumulated intermediate steps can have dramatic effect on the rapid synchronization of the network as a whole. In other words, complex problem-solving networks will synchronize much more rapidly if there are synchronizable intermediate configurations than if there are not. It is thus plausible, as our results also suggest, that problem-solving networks are biased towards subgraphs in which it is easier to synchronized.

As was shown in Fig. 3, subgraphs that include feedback loops (e.g., the regulated mutual, semi-clique, and clique subgraphs) are less likely to synchronize. We therefore expect that these feedback-based subgraphs will be underrepresented in the networks (when compared with random networks). However, our study reveals that, while being relatively rare, these subgraphs are sometimes overrepresented in the networks. This suggests that other factors and selective pressures, besides synchronizability, are important in determining the prevalence of network subgraphs. One possible explanation might be that high performance of problem-solving networks usually involves a trade-off between the exploration for new information that could impede effective synchronization, and the exploitation of existing knowledge that facilitates effective synchronization. It is thus plausible that the abundance of highly-synchronizable subgraphs combined with the existence of subgraphs that include feedback loops reflect the balance between these exploratory and exploitative activities in real problem-solving networks.

For small-scale problem-solving networks, it is possible to generate highly optimizable interconnections that promote synchronization among group members since linking constraints are easy to satisfy. As the complexity of problem-solving crosses over some threshold, the purposeful attainment of highly-synchronizable structures becomes more difficult. In such cases, it is possible that the observed diversity of subgraphs in problem-solving networks reflects the cumulative effect of various short-term adaptive processes that combine to produce change in the local network structure over long-periods of time. The network structure of the problem-solving activity is modified to meet changes in the requirements of previously implemented solutions. For example, starting from previous group interactions underlying vehicle development the problem-solving network will change organically as incremental or innovative changes occur at the subsystem and component levels of the car. Similar considerations apply to many other problem-solving processes including drug development, economic development, or social planning. In each problem-solving phase, various group interactions are attempted, their consequences are observed, and this information is used to guide the “re-wiring” of group interactions. The results in this paper might suggest that one of the forces behind the link rearrangement of group interactions is adaptation, present at different levels, towards effective synchronization within constraints of the problem-solving activity. The evolution of problem-solving networks shares some basic characteristics with evolutionary change in biotic populations, which involves a process of variation, selection, and transmission. If a collection of organizational patterns (subgraphs) differ in the efficiency with which they can synchronize, selection processes will favor such patterns of ties. Over time, such selection processes will lead to increased abundance of the more fit patterns of ties. The results in this paper suggest that selectivity of synchronizable configurations may guide the evolution of the problem-solving network, a mechanism that is essential for its subsequent rapidity. Changes in the structure of organizational interconnections emerge not only through selection, but also through a variety of transmission mechanisms, including mimicry, copying, learning, and re-use of network motifs^1,2^. Organizations that are involved in problem-solving activities attempt to implement ‘best practices’ or adopt organizational forms extracted from other problem-solving activities that were successful in the past^77^. Such imitation or copying processes would give rise to an increasing number of synchronizable network forms of organization.

The similarity in the local structure of distinct large-scale problem-solving networks, based on their subgraph significance and relative difference profiles (Figs. 9 and 15), leads to the intriguing possibility that subgraph abundance in problem-solving networks is a manifestation of organizational “routines.” The concept of routines plays a key role in organizational theory, strategic management, and evolutionary economics^78–80^. Routines are defined as recurrent patterns of interactions executed by various actors, which represent behaviors, knowledge, or capabilities held in organizational memory^79,81^. Organizational routines were proposed as analogous to biological genes^79^ in which they are passed on by various transmission processes, such as learning and copying. Our theory could provide a bridge between the science of complex networks and the well-established concept of organizational routines. In particular, the study of network subgraphs could provide a powerful perspective on organizational routines. Our results show that large-scale problem-solving networks share repeated patterns of interdependent activities (routines) that are not idiosyncratic to a specific problem-solving organization but are universal across many distinct organizations. These repeated patterns might then be considered as problem-solving routines—patterns of activity ties occurring in the network at numbers that are significantly higher than those in randomized networks, and that are conserved across a large number of distinct problem-solving networks. The main result in this paper is that the abundance of these problem-solving routines is highly correlated with their ability to synchronize and coordinate the problem-solving activity.

In conclusion, the results in this paper reveal a strong association between a dynamic property of network subgraphs—synchronizability—and the frequency and significance of these subgraphs in real-world problem-solving networks. We suggested the possibility that selective pressures that favor more synchronizable subgraphs could account for the diverse abundance of subgraphs in problem-solving networks. Our empirical results show that unrelated problem-solving networks display very similar local network structure, defined in terms of the significance and relative difference profiles of three- and four-nodes connected subgraphs. These observations led us to the hypothesis that network subgraphs represent organizational routines that enable better coordination and control of the problem-solving activity as well as the exchange and sharing of knowledge within and across problem-solving activities.

## Supplementary information

Supplementary Information
