# Supplementary material for: Patterns of ties in problem-solving networks and their dynamic properties
Source: Sci Rep. 2020 Oct 22;10:18137. doi: 10.1038/s41598-020-75221-3 (PMC7582982; doi:10.1038/s41598-020-75221-3)
Supplement: Supplementary file 1 — Supplementary Information [file 41598_2020_75221_MOESM1_ESM.docx]

**Patterns of Ties in Problem-Solving Networks and their Dynamic Properties**

Dan Braha^a,b,*^

^a^ New England Complex Systems Institute, Cambridge, MA, United States,

^b^ University of Massachusetts Dartmouth, Dartmouth, MA, United States

^*^ Corresponding author

Prof. Dan Braha, New England Complex Systems Institute, 277 Broadway, Cambridge, MA 02139, USA.

Tel: (617)5992906

E-mail address: braha@necsi.edu

Supplementary Data

Here, we provide information related to all of the problem-solving networks analyzed in the paper: Vehicle Problem-Solving Network (‘Veh1,’ Page 2 below), Real Estate Problem-Solving Network (‘Red,’ Page 25 below), Strategy and Knowledge Problem-Solving Network (‘Knwl,’ Page 89 below), Microprocessor Problem-Solving Network (‘Mip,’ Page 105 below), Bioscience Facility Problem-Solving Network (‘Bio,’ Page 122 below), Vehicle Problem-Solving Network (‘Veh2,’ Page 135 below), and Equipment Problem-Solving Network (‘Equip,’ Page 149 below).

The networks are represented as a list of directed links (edges) using the following format:

<source node> <target node> <edge weight>

Nodes in the network are represented by integers. For all networks, the <edge weight> is 1 for all edges. For example,

1 2 1

2 3 1

represents a network of 3 nodes with two directed links:

**1**

**2**

**3**

**Supplementary Data S1. Vehicle Problem-Solving Network (‘Veh1’)**

The ‘Veh1’ problem-solving network includes 120 nodes and 417 directed links, and is represented as follows:

1 3 1

1 5 1

1 6 1

1 7 1

1 8 1

1 10 1

1 11 1

1 12 1

1 13 1

1 14 1

1 15 1

1 16 1

1 18 1

1 20 1

1 21 1

1 22 1

1 25 1

1 30 1

1 39 1

1 40 1

1 41 1

1 42 1

1 43 1

1 44 1

2 51 1

2 52 1

2 53 1

2 54 1

3 16 1

3 18 1

3 20 1

3 22 1

3 25 1

3 29 1

3 30 1

3 37 1

3 80 1

3 81 1

4 39 1

4 91 1

4 118 1

5 9 1

6 7 1

7 14 1

7 18 1

7 20 1

7 35 1

7 80 1

7 115 1

8 13 1

8 36 1

9 97 1

10 11 1

10 28 1

10 40 1

10 41 1

10 42 1

10 43 1

10 44 1

10 65 1

10 97 1

10 112 1

11 12 1

11 19 1

11 25 1

11 28 1

11 30 1

11 51 1

11 52 1

11 53 1

11 54 1

11 65 1

11 97 1

11 112 1

12 15 1

12 38 1

13 14 1

13 17 1

14 13 1

14 35 1

15 38 1

15 55 1

16 17 1

16 19 1

16 40 1

16 41 1

16 42 1

16 43 1

16 80 1

16 109 1

17 20 1

17 33 1

17 35 1

17 63 1

17 80 1

17 81 1

17 107 1

17 109 1

17 112 1

18 19 1

18 30 1

18 37 1

18 39 1

19 20 1

19 37 1

20 35 1

20 89 1

21 23 1

22 34 1

23 24 1

24 23 1

25 26 1

25 27 1

25 37 1

25 71 1

26 27 1

26 31 1

26 56 1

27 17 1

27 35 1

27 56 1

27 76 1

27 77 1

27 80 1

27 81 1

27 98 1

27 109 1

28 29 1

28 49 1

29 37 1

29 39 1

30 28 1

30 31 1

30 32 1

30 37 1

31 32 1

32 31 1

32 67 1

32 68 1

32 74 1

32 84 1

33 35 1

34 54 1

34 64 1

34 70 1

34 99 1

35 39 1

35 89 1

36 39 1

36 90 1

37 27 1

37 34 1

37 38 1

37 39 1

37 40 1

37 41 1

37 42 1

37 43 1

37 44 1

37 63 1

37 65 1

38 29 1

38 55 1

39 4 1

39 37 1

39 40 1

39 41 1

39 42 1

39 43 1

40 45 1

40 51 1

40 73 1

40 87 1

40 92 1

41 46 1

41 52 1

41 73 1

41 87 1

41 92 1

42 47 1

42 53 1

42 73 1

42 87 1

42 92 1

43 48 1

43 54 1

43 73 1

43 87 1

43 92 1

44 24 1

44 34 1

44 50 1

44 54 1

44 99 1

45 65 1

45 72 1

45 92 1

45 93 1

46 65 1

46 72 1

46 92 1

46 94 1

47 65 1

47 72 1

47 92 1

47 95 1

48 65 1

48 72 1

48 92 1

48 96 1

49 65 1

49 92 1

50 48 1

50 99 1

51 57 1

51 66 1

51 100 1

52 58 1

52 67 1

52 101 1

53 59 1

53 68 1

53 102 1

54 34 1

54 60 1

54 69 1

54 103 1

55 87 1

56 65 1

56 71 1

56 82 1

56 84 1

57 61 1

57 62 1

58 32 1

58 61 1

58 62 1

58 67 1

59 32 1

59 61 1

59 62 1

59 68 1

60 61 1

60 62 1

61 66 1

61 67 1

61 68 1

61 69 1

61 71 1

61 83 1

61 84 1

61 104 1

61 106 1

62 65 1

62 82 1

62 83 1

62 84 1

62 112 1

63 71 1

63 93 1

63 94 1

63 95 1

63 96 1

63 109 1

64 65 1

65 74 1

65 90 1

65 91 1

65 93 1

65 94 1

65 95 1

65 96 1

65 112 1

66 73 1

66 91 1

66 93 1

67 73 1

67 91 1

67 94 1

68 73 1

68 91 1

68 95 1

69 70 1

69 73 1

69 91 1

69 96 1

70 50 1

70 91 1

70 96 1

70 99 1

71 73 1

71 91 1

71 98 1

72 45 1

72 46 1

72 47 1

72 48 1

72 73 1

72 79 1

72 84 1

72 85 1

72 86 1

72 90 1

72 93 1

72 94 1

72 95 1

72 96 1

72 112 1

73 80 1

73 81 1

73 88 1

73 89 1

73 107 1

73 109 1

73 110 1

73 117 1

73 119 1

74 75 1

74 90 1

75 91 1

75 93 1

75 94 1

75 95 1

75 96 1

75 118 1

76 49 1

76 77 1

77 71 1

77 76 1

77 78 1

78 89 1

79 72 1

79 89 1

80 93 1

80 94 1

80 95 1

80 96 1

81 93 1

81 94 1

81 95 1

81 96 1

82 106 1

83 77 1

83 84 1

84 106 1

84 110 1

84 112 1

84 116 1

86 73 1

87 65 1

88 89 1

89 91 1

89 115 1

89 117 1

89 118 1

90 91 1

90 117 1

90 118 1

90 119 1

91 4 1

91 112 1

92 105 1

92 120 1

93 72 1

93 84 1

93 100 1

94 72 1

94 84 1

94 101 1

95 72 1

95 84 1

95 102 1

96 72 1

96 84 1

96 103 1

97 72 1

98 73 1

98 76 1

98 77 1

98 92 1

98 106 1

99 103 1

100 84 1

100 116 1

101 84 1

101 116 1

102 84 1

102 116 1

103 84 1

103 99 1

103 104 1

103 116 1

104 111 1

104 116 1

105 112 1

106 107 1

106 108 1

106 110 1

106 112 1

106 116 1

107 108 1

108 109 1

109 110 1

110 113 1

112 72 1

112 97 1

112 118 1

113 114 1

114 118 1

115 89 1

115 118 1

116 111 1

116 118 1

116 119 1

118 4 1

118 117 1

118 119 1

**Supplementary Data 2. Real Estate Problem-Solving Network (‘Red’)**

The ‘Red’ problem-solving network includes 91 nodes and 1148 directed links, and is represented as follows:

1 2 1

1 3 1

1 7 1

1 11 2

1 12 2

1 13 2

1 14 2

1 15 2

1 16 2

1 18 1

1 28 2

1 29 1

1 30 2

1 31 2

1 32 2

1 37 1

1 38 1

1 50 1

1 52 2

1 53 2

1 56 2

1 57 2

1 58 2

1 60 2

1 62 1

1 67 2

1 77 2

1 78 1

1 83 1

1 88 1

1 91 1

2 1 1

2 3 1

2 7 1

2 8 2

2 9 2

2 10 2

2 11 2

2 12 2

2 13 2

2 14 2

2 16 2

2 18 1

2 20 2

2 29 1

2 30 2

2 31 2

2 32 2

2 37 1

2 38 1

2 52 2

2 53 2

2 56 2

2 57 2

2 58 2

2 62 1

2 65 2

2 77 2

2 78 1

2 83 1

2 88 1

2 89 2

2 91 1

3 6 1

3 7 1

3 8 2

3 10 2

3 12 2

3 13 2

3 14 2

3 15 2

3 16 2

3 18 1

3 19 1

3 27 2

3 28 2

3 29 1

3 32 2

3 37 1

3 38 1

3 39 2

3 40 2

3 50 1

3 53 2

3 56 2

3 57 2

3 58 2

3 60 2

3 61 2

3 62 1

3 67 2

3 77 2

3 78 1

3 83 1

3 88 1

3 91 1

4 5 1

4 7 2

4 8 2

4 9 2

4 12 2

4 13 2

4 14 2

4 15 2

4 18 2

4 20 2

4 21 1

4 22 2

4 23 2

4 24 1

4 25 1

4 27 2

4 29 2

4 44 1

4 45 1

5 4 1

5 7 2

5 8 2

5 9 2

5 12 2

5 13 2

5 14 2

5 15 2

5 20 2

5 21 1

5 23 2

5 24 1

5 25 1

5 27 2

5 29 2

5 44 1

5 45 1

5 49 1

6 7 1

6 10 2

6 14 2

6 15 2

6 16 2

6 18 1

6 19 1

6 29 1

6 30 2

6 31 2

6 32 2

6 37 1

6 38 1

7 6 1

7 10 2

7 14 2

7 15 2

7 16 2

7 18 1

7 19 1

7 23 2

7 25 2

7 27 2

7 28 2

7 29 1

7 30 2

7 31 2

7 32 2

7 37 1

7 38 1

7 39 2

8 4 2

8 5 2

8 7 2

8 14 2

8 15 2

8 16 2

8 18 2

8 20 2

8 21 2

8 22 2

8 23 2

8 24 2

8 25 2

8 26 2

8 27 2

8 28 2

8 29 2

8 30 2

8 31 2

8 32 2

8 35 1

8 66 1

9 8 1

9 10 2

9 12 1

9 13 1

9 16 2

9 20 2

9 21 2

9 22 2

9 29 2

9 32 2

9 35 1

9 66 1

10 6 2

10 7 2

10 11 1

10 12 2

10 14 2

10 15 2

10 16 1

11 10 1

11 12 2

11 14 2

11 15 2

11 16 1

11 29 2

11 30 1

11 31 1

11 32 1

11 52 1

11 53 1

12 5 2

12 7 2

12 8 1

12 9 1

12 11 2

12 14 2

12 15 2

12 16 2

12 17 2

12 19 2

12 20 2

12 21 2

12 23 2

12 27 2

12 29 2

12 30 2

12 31 2

12 32 2

12 33 1

13 7 2

13 8 1

13 9 1

13 10 2

13 12 1

13 14 2

13 15 2

13 16 2

13 17 2

13 18 2

13 19 2

13 20 2

13 21 2

13 22 2

13 23 2

13 24 2

13 25 2

13 27 2

13 28 2

13 29 2

13 30 2

13 31 2

13 32 2

13 34 1

14 7 2

14 8 2

14 10 2

14 12 2

14 13 2

14 15 1

14 16 2

14 18 2

14 19 2

14 20 1

14 21 2

14 22 1

14 23 1

14 24 2

14 25 2

14 26 1

14 27 1

14 28 1

14 29 2

14 30 2

14 31 2

14 32 2

15 6 2

15 7 2

15 8 2

15 10 2

15 12 2

15 13 2

15 14 1

15 16 2

15 18 2

15 19 2

15 20 1

15 21 2

15 22 1

15 23 1

15 24 2

15 25 2

15 26 1

15 27 1

15 28 1

15 29 2

15 30 2

15 31 2

15 32 2

16 6 2

16 7 2

16 11 1

16 14 2

16 15 2

16 17 2

16 18 2

16 19 2

16 20 2

16 21 2

16 22 2

16 23 2

16 26 2

16 28 2

16 29 2

16 30 1

16 31 1

16 32 1

17 18 2

17 19 2

17 20 2

17 21 2

17 22 2

17 23 2

17 24 2

17 25 2

17 26 2

17 27 2

17 28 2

17 29 2

17 30 2

17 31 2

17 32 2

17 33 2

17 34 2

17 35 2

18 19 1

18 22 2

18 24 2

18 28 2

18 29 1

18 32 2

18 33 2

18 34 2

18 35 2

18 37 1

18 38 1

19 22 2

19 23 2

19 24 2

19 27 2

19 28 2

19 29 1

19 32 2

19 33 2

19 34 2

19 35 2

19 37 1

19 38 1

20 18 2

20 19 2

20 21 2

20 22 1

20 23 1

20 24 2

20 25 2

20 26 1

20 27 1

20 28 1

20 29 2

20 32 2

20 33 2

20 34 2

20 39 1

20 40 1

21 18 2

21 20 2

21 22 2

21 23 2

21 24 1

21 25 1

21 26 2

21 27 2

21 29 2

21 32 2

21 33 2

21 34 2

21 35 2

21 40 2

21 44 1

21 45 1

21 49 1

22 18 2

22 19 2

22 23 1

22 25 2

22 26 1

22 27 1

22 28 1

22 29 2

22 30 2

22 31 2

22 32 2

22 33 2

22 34 2

22 35 2

22 37 2

22 38 2

22 39 1

22 40 1

22 42 1

22 44 2

22 45 2

22 46 1

22 47 1

23 18 2

23 19 2

23 22 1

23 27 1

23 28 1

23 29 2

23 32 2

23 33 2

23 34 2

23 35 2

23 37 2

23 38 2

23 39 1

23 40 1

23 42 1

23 44 2

23 45 2

23 46 1

23 47 1

24 19 2

24 20 2

24 21 1

24 23 2

24 25 1

24 26 2

24 27 2

24 32 2

24 33 2

24 34 2

24 35 2

24 38 2

24 40 2

24 44 1

24 45 1

24 47 2

24 49 1

25 20 2

25 21 1

25 22 2

25 23 2

25 24 1

25 26 2

25 27 2

25 28 2

25 29 2

25 32 2

25 33 2

25 34 2

25 35 2

25 39 2

25 40 2

25 44 1

25 45 1

25 46 2

25 47 2

25 49 1

26 24 2

26 25 2

26 28 1

26 29 2

26 32 2

26 33 2

26 34 2

26 35 2

26 39 1

26 40 1

26 42 1

26 44 2

26 45 2

26 46 1

26 47 1

26 49 2

27 25 2

27 28 1

27 32 2

27 33 2

27 34 2

27 38 2

27 39 1

27 40 1

27 42 1

28 23 1

28 29 2

28 30 2

28 31 2

28 32 2

28 33 2

28 34 2

28 35 2

28 39 1

28 40 1

28 42 1

29 22 2

29 23 2

29 27 2

29 28 2

29 30 2

29 31 2

29 32 2

29 33 2

29 34 2

29 35 2

29 37 1

29 38 1

29 39 2

29 40 2

29 42 2

29 44 2

29 45 2

29 46 2

29 47 2

30 29 2

30 31 1

30 32 1

30 33 2

30 34 2

30 35 2

30 38 2

30 50 2

30 52 1

30 53 1

30 56 1

30 57 1

30 58 1

31 29 2

31 30 1

31 32 1

31 33 2

31 34 2

31 35 2

31 38 2

31 50 2

31 52 1

31 53 1

31 56 1

31 57 1

31 58 1

32 21 2

32 22 2

32 23 2

32 28 2

32 29 2

32 30 1

32 31 1

32 33 2

32 34 2

32 35 2

32 36 2

32 38 2

32 39 2

32 50 2

32 52 1

32 56 1

32 57 1

33 21 2

33 24 2

33 27 2

33 28 2

33 29 2

33 30 2

33 31 2

33 32 2

33 34 1

33 35 1

33 36 2

33 37 2

33 38 2

33 39 2

33 40 2

33 42 2

33 44 2

33 45 2

33 46 2

33 47 2

33 49 2

33 50 2

33 52 2

33 53 2

33 55 2

33 60 2

33 61 1

34 27 2

34 28 2

34 29 2

34 30 2

34 31 2

34 32 2

34 33 1

34 35 1

34 36 2

34 37 2

34 38 2

34 39 2

34 40 2

34 42 2

34 44 2

34 45 2

34 47 2

34 49 2

34 50 2

34 52 2

34 53 2

34 55 2

34 60 2

34 61 1

35 34 1

35 36 2

35 37 2

35 38 2

35 39 2

35 40 2

35 42 2

35 43 2

35 44 2

35 45 2

35 46 2

35 47 2

35 49 2

35 50 2

35 52 2

35 53 2

35 55 2

35 60 2

35 61 1

35 66 1

36 37 2

36 38 2

36 39 2

36 40 2

36 41 2

36 42 2

36 43 2

36 44 2

36 45 2

36 46 2

36 47 2

36 48 2

36 49 2

36 50 2

36 51 2

36 52 2

36 53 2

36 54 2

36 55 2

36 56 2

36 57 2

36 58 2

36 59 2

36 60 2

36 61 2

36 62 2

37 38 1

37 40 2

37 45 2

37 50 1

37 52 2

37 55 1

37 56 2

37 57 2

37 58 2

37 59 2

37 61 2

37 62 1

37 65 2

37 78 1

38 37 1

38 40 2

38 45 2

38 47 2

38 50 1

38 52 2

38 53 2

38 55 1

38 56 2

38 57 2

38 58 2

38 59 2

38 61 2

38 62 1

38 65 2

39 40 1

39 41 1

39 42 1

39 61 2

39 64 1

40 42 1

40 43 2

40 44 2

40 45 2

40 46 1

40 47 1

40 48 1

40 49 2

40 50 2

40 51 1

40 52 2

40 54 1

40 56 2

40 57 2

40 59 1

40 60 1

40 61 2

40 62 2

40 65 2

40 67 1

41 42 1

41 51 1

41 52 2

41 56 2

41 57 2

41 60 1

41 61 2

41 64 1

41 67 1

42 44 2

42 45 2

42 46 1

42 47 1

42 48 1

42 49 2

42 50 2

42 51 1

42 52 2

42 54 1

42 55 2

42 56 2

42 57 2

42 59 1

42 60 1

42 61 2

42 62 2

42 64 1

42 65 2

42 67 1

43 46 2

43 52 2

43 56 2

43 57 2

43 61 2

43 63 2

43 65 2

43 66 2

44 45 1

44 46 2

44 47 2

44 48 2

44 49 1

44 56 2

44 57 2

44 61 2

45 48 2

45 49 1

45 51 2

45 54 2

45 56 2

45 57 2

45 61 2

45 63 2

45 65 2

45 66 2

45 68 1

46 42 1

46 45 2

46 49 2

46 51 1

46 52 2

46 56 2

46 57 2

46 61 2

46 65 2

46 66 2

46 68 2

47 42 1

47 44 2

47 45 2

47 48 1

47 49 2

47 50 2

47 51 1

47 52 2

47 54 1

47 55 2

47 56 2

47 57 2

47 59 1

47 60 1

47 61 2

47 62 2

48 42 1

48 44 2

48 45 2

48 49 2

48 51 1

48 54 1

48 55 2

48 56 2

48 57 2

48 59 1

48 60 1

48 61 2

49 43 1

49 44 1

49 45 1

49 48 2

49 56 2

49 57 2

49 61 2

49 63 2

49 68 1

50 44 2

50 52 2

50 56 2

50 57 2

50 58 2

50 59 2

50 61 2

50 62 1

50 78 1

50 80 2

51 42 1

51 49 2

51 54 1

51 56 2

51 57 2

51 60 1

51 61 2

51 64 1

51 67 1

51 68 2

51 69 1

51 70 1

51 72 1

51 73 2

52 53 1

52 56 1

52 57 1

52 58 1

52 61 2

52 65 1

52 66 2

52 77 1

53 56 1

53 57 1

53 61 2

53 65 1

53 77 1

54 42 1

54 56 2

54 57 2

54 60 1

54 61 2

54 63 2

54 64 1

54 65 2

54 69 1

54 70 1

54 71 1

54 72 1

54 73 2

55 54 2

55 56 2

55 57 2

55 59 2

55 60 2

55 61 2

55 62 1

55 71 2

55 77 2

55 78 1

55 80 2

56 39 2

56 40 2

56 42 2

56 44 2

56 45 2

56 47 2

56 48 2

56 50 2

56 51 2

56 52 1

56 53 1

56 54 2

56 57 1

56 58 1

56 59 2

56 60 2

56 61 2

56 62 2

56 64 2

56 65 1

56 66 2

56 76 1

56 77 1

57 39 2

57 40 2

57 42 2

57 44 2

57 45 2

57 47 2

57 48 2

57 50 2

57 51 2

57 52 1

57 53 1

57 54 2

57 55 2

57 56 1

57 58 1

57 59 2

57 61 2

57 62 2

57 63 2

57 65 1

57 66 2

57 77 1

58 40 2

58 41 2

58 44 2

58 45 2

58 47 2

58 48 2

58 51 2

58 52 1

58 53 1

58 54 2

58 55 2

58 56 1

58 57 1

58 61 2

58 62 2

58 65 1

58 66 2

58 77 1

59 42 1

59 50 2

59 51 1

59 54 1

59 55 2

59 56 2

59 57 2

59 61 2

59 62 2

59 64 1

59 77 2

60 42 1

60 51 1

60 54 1

60 56 2

60 57 2

60 59 1

60 61 2

60 64 1

60 67 1

61 42 2

61 44 2

61 47 2

61 48 2

61 50 2

61 51 2

61 52 2

61 54 2

61 55 2

61 56 2

61 57 2

61 58 2

61 59 2

61 60 2

61 62 2

61 63 2

61 65 2

61 66 1

61 67 2

61 68 2

61 69 2

61 70 2

61 71 2

61 77 2

61 79 1

62 51 2

62 54 2

62 55 1

62 56 2

62 57 2

62 58 2

62 61 2

62 63 2

62 65 2

62 71 2

62 77 2

62 78 1

62 80 2

63 64 2

63 65 2

63 66 2

63 67 2

63 68 2

63 69 2

63 70 2

63 71 2

63 72 2

63 73 2

63 74 2

63 75 2

63 76 2

63 77 2

63 78 2

63 79 2

64 65 2

64 67 1

64 68 2

64 69 1

64 70 1

64 71 1

64 76 2

64 77 2

64 79 2

65 66 2

65 67 2

65 68 2

65 69 2

65 70 2

65 71 2

65 73 1

65 76 1

65 77 1

65 79 2

65 89 1

66 68 2

66 69 2

66 70 2

66 71 2

66 76 2

66 77 2

66 79 1

67 69 1

67 70 1

67 71 1

67 76 2

67 77 2

67 79 2

68 69 2

68 70 2

68 71 2

68 79 2

69 70 1

69 71 1

69 72 1

69 73 2

69 74 2

69 75 1

69 76 2

69 79 2

69 80 1

69 84 1

70 71 1

70 72 1

70 73 2

70 74 2

70 75 1

70 76 2

70 79 2

70 80 1

70 84 1

71 72 1

71 73 2

71 76 2

71 79 2

71 80 1

71 84 1

72 73 2

72 74 2

72 75 1

72 76 2

72 79 2

72 84 1

73 79 2

73 85 1

73 87 2

74 75 2

74 79 2

74 80 2

74 82 2

74 86 1

74 89 2

75 79 2

75 80 1

75 82 2

76 77 1

76 79 2

77 76 1

77 79 2

77 85 1

78 76 2

78 79 2

78 83 1

78 88 1

79 76 2

79 78 2

79 82 1

79 83 2

79 85 2

79 88 2

80 81 2

80 83 2

80 85 2

80 89 2

81 83 2

81 85 1

81 89 1

82 84 2

82 85 2

82 90 1

83 85 2

83 88 1

83 89 2

84 86 2

84 87 1

85 88 2

85 89 1

85 90 2

86 87 2

87 90 2

88 89 2

88 90 2

88 91 1

89 90 2

89 91 2

90 89 2

90 91 2

**Supplementary Data 3.** Strategy and Knowledge Problem-Solving Network (‘Knwl’)

The ‘Knwl’ problem-solving network includes 62 nodes and 285 directed links, and is represented as follows:

1 6 1

1 7 1

1 9 1

2 3 1

2 4 1

2 5 1

2 6 1

2 9 1

3 4 1

3 5 1

3 9 1

3 57 1

4 3 1

4 5 1

4 6 1

4 9 1

4 29 1

5 3 1

5 4 1

5 6 1

5 9 1

6 19 1

6 25 1

6 29 1

7 8 1

7 9 1

8 9 1

8 12 1

8 25 1

9 7 1

9 8 1

9 10 1

9 11 1

9 12 1

10 8 1

10 11 1

10 12 1

10 16 1

10 17 1

10 18 1

10 20 1

10 23 1

10 25 1

10 30 1

10 46 1

10 47 1

10 53 1

11 60 1

11 62 1

12 13 1

12 14 1

12 15 1

12 16 1

12 17 1

12 25 1

12 29 1

12 34 1

12 47 1

12 50 1

12 60 1

13 16 1

13 17 1

13 30 1

13 34 1

14 30 1

15 16 1

15 17 1

15 25 1

15 27 1

15 28 1

15 29 1

15 30 1

15 44 1

18 19 1

18 20 1

18 21 1

18 22 1

18 24 1

18 25 1

18 29 1

18 41 1

18 53 1

19 20 1

19 21 1

19 25 1

19 29 1

20 18 1

20 19 1

20 21 1

20 25 1

20 29 1

20 39 1

20 45 1

21 22 1

21 23 1

21 24 1

21 25 1

21 26 1

21 29 1

21 32 1

21 37 1

21 40 1

21 41 1

21 43 1

21 44 1

21 45 1

21 46 1

21 47 1

21 48 1

21 50 1

21 53 1

21 59 1

22 18 1

22 25 1

22 32 1

22 35 1

22 36 1

22 39 1

22 40 1

22 41 1

22 45 1

22 46 1

22 52 1

22 59 1

23 48 1

23 49 1

23 51 1

24 26 1

24 48 1

24 49 1

24 50 1

24 51 1

25 27 1

25 28 1

25 29 1

25 30 1

26 36 1

26 40 1

26 48 1

26 49 1

26 51 1

26 53 1

26 59 1

27 29 1

27 30 1

28 29 1

28 30 1

31 39 1

31 45 1

31 46 1

31 57 1

31 59 1

31 60 1

32 36 1

32 39 1

32 45 1

32 46 1

32 52 1

32 59 1

32 60 1

33 34 1

33 41 1

34 36 1

34 57 1

34 60 1

35 32 1

35 34 1

35 36 1

35 59 1

35 60 1

36 34 1

36 51 1

36 60 1

37 38 1

38 36 1

38 39 1

38 51 1

38 52 1

38 57 1

38 58 1

38 59 1

38 60 1

38 62 1

39 32 1

39 45 1

39 47 1

39 58 1

40 31 1

40 32 1

40 37 1

40 41 1

40 44 1

40 46 1

40 52 1

40 58 1

41 31 1

41 33 1

41 37 1

41 42 1

41 43 1

41 44 1

41 45 1

41 46 1

42 31 1

42 32 1

42 45 1

42 46 1

42 47 1

42 58 1

43 31 1

43 32 1

43 37 1

43 44 1

43 45 1

43 46 1

43 47 1

43 58 1

44 31 1

44 32 1

44 37 1

44 41 1

44 45 1

44 46 1

44 47 1

44 48 1

44 52 1

44 58 1

44 59 1

44 62 1

45 31 1

45 32 1

45 37 1

45 47 1

45 58 1

46 31 1

46 32 1

46 37 1

46 45 1

46 47 1

46 58 1

47 31 1

47 32 1

47 44 1

47 46 1

47 58 1

48 49 1

48 50 1

48 51 1

49 51 1

50 51 1

51 34 1

51 36 1

51 40 1

51 53 1

51 55 1

51 59 1

51 60 1

51 62 1

52 32 1

52 35 1

52 36 1

52 59 1

53 36 1

53 54 1

53 55 1

53 56 1

53 59 1

54 55 1

54 56 1

54 59 1

55 34 1

55 56 1

55 59 1

56 34 1

56 36 1

56 59 1

56 60 1

56 62 1

57 60 1

57 62 1

58 60 1

58 62 1

60 61 1

60 62 1

61 62 1

**Supplementary Data 4. Microprocessor Problem-Solving Network (‘Mip’)**

The ‘Mip’ problem-solving network includes 60 nodes and 301 directed links, and is represented as follows:

1 2 2

1 3 2

1 6 1

1 9 1

1 10 1

1 11 1

1 46 1

1 47 1

1 48 1

2 7 1

2 11 1

2 13 1

2 38 1

2 44 1

2 45 1

2 46 1

2 47 1

3 1 2

3 2 2

3 6 1

3 7 1

3 13 1

3 38 1

3 46 1

3 47 1

4 1 2

4 2 2

4 3 2

4 5 2

4 7 1

4 13 1

4 14 1

4 15 1

4 46 1

4 52 1

4 53 1

4 59 1

5 4 2

5 7 2

5 12 1

5 14 1

5 15 1

5 59 1

6 5 2

6 7 2

6 8 2

6 9 2

6 10 2

6 12 1

6 13 1

6 16 1

6 18 1

6 19 1

6 20 1

7 6 2

7 9 2

8 5 2

8 6 2

8 9 2

8 10 2

8 11 1

8 13 1

8 16 1

8 17 1

8 18 1

8 19 1

8 20 1

8 24 1

9 5 2

9 6 2

9 8 2

9 10 2

9 11 1

9 13 1

9 18 1

9 19 1

9 27 1

9 29 1

10 5 2

10 6 2

10 11 2

10 12 1

10 13 1

10 14 1

10 16 1

10 18 1

10 19 1

10 20 1

10 38 1

10 44 1

10 60 1

11 10 2

11 12 1

11 13 1

11 14 1

11 16 1

11 17 1

11 18 1

11 19 1

11 20 1

11 21 1

11 23 1

11 27 1

11 29 1

11 41 1

11 48 1

11 57 1

12 13 2

12 14 2

12 15 2

12 17 1

12 18 1

12 19 1

12 20 1

12 22 1

12 27 1

13 12 2

13 18 1

13 19 1

13 29 1

14 12 2

14 13 2

14 15 2

14 16 1

14 17 1

14 21 1

14 22 1

14 23 1

15 12 2

15 13 2

15 14 2

15 16 1

15 24 1

15 25 1

15 43 1

16 17 1

16 18 1

16 19 1

16 20 1

17 18 2

17 22 2

17 24 2

18 17 2

18 19 2

18 22 2

18 26 1

19 17 2

19 20 2

19 22 2

19 26 1

20 17 2

20 22 2

20 23 2

20 24 2

21 17 2

21 20 2

21 22 2

21 23 2

21 24 2

21 25 2

21 42 1

21 49 1

22 17 2

22 21 2

22 23 2

22 24 2

22 25 2

22 27 1

22 28 1

22 34 1

22 35 1

22 37 1

22 42 1

23 17 2

23 22 2

23 24 2

23 25 2

23 26 2

23 27 1

23 28 1

23 29 1

23 30 1

23 31 1

23 49 1

24 17 2

24 20 2

24 21 2

24 22 2

24 25 2

24 26 2

25 17 2

25 20 2

25 21 2

25 22 2

25 23 2

25 24 2

25 26 2

25 35 1

26 23 2

26 24 2

26 25 2

26 27 2

26 28 2

26 29 1

26 30 1

26 31 1

27 26 2

27 30 1

28 26 2

28 30 1

29 36 1

29 41 1

29 49 1

30 31 2

31 30 2

31 32 1

32 33 2

32 35 2

33 35 2

33 36 1

34 35 2

34 37 1

35 32 2

35 36 1

36 37 1

36 41 1

36 48 1

37 38 1

37 41 1

37 42 1

37 43 1

38 39 1

39 40 1

39 48 1

40 42 1

40 43 1

40 57 1

41 43 1

41 50 1

41 51 1

41 57 1

42 43 1

42 49 1

42 50 1

42 51 1

42 57 1

43 49 1

43 53 1

44 45 2

44 46 2

44 48 2

44 50 1

44 51 1

45 44 2

45 48 2

45 59 1

45 60 1

46 44 2

46 59 1

46 60 1

47 46 2

47 59 1

47 60 1

48 45 2

48 59 1

48 60 1

49 59 1

49 60 1

50 51 2

50 52 2

50 54 1

50 56 1

50 57 1

51 50 2

51 52 2

51 54 1

51 56 1

51 57 1

52 50 2

52 51 2

52 54 1

52 55 1

52 57 1

52 58 1

53 56 1

53 57 1

53 59 1

53 60 1

54 56 1

54 57 1

55 56 2

55 57 2

55 58 1

56 55 2

56 57 2

56 58 1

57 55 2

57 56 2

57 60 1

59 60 2

60 58 2

**Supplementary Data 5. Bioscience Facility Problem-Solving Network (‘Bio’)**

The ‘Bio’ problem-solving network includes 53 nodes and 230 directed links, and is represented as follows:

1 13 3

1 20 3

1 23 3

1 24 3

1 29 3

2 11 3

2 13 3

2 24 3

2 27 3

2 39 3

3 14 3

3 16 3

3 17 3

3 18 3

3 21 3

3 22 3

3 26 3

3 28 3

3 32 3

3 37 3

3 39 3

3 42 3

3 43 3

3 44 3

3 48 3

4 14 3

4 20 3

4 24 2

4 25 3

4 28 3

4 29 3

4 30 3

4 31 3

4 32 3

4 33 3

4 34 2

4 42 3

5 28 3

5 29 3

5 31 3

5 32 3

5 35 2

5 42 3

6 16 3

6 20 3

6 42 3

6 43 3

7 15 3

7 27 3

7 29 3

7 39 3

7 43 3

7 45 3

8 15 3

8 16 3

8 39 3

8 43 3

8 45 3

9 51 3

10 12 3

10 16 3

10 18 3

10 19 3

10 25 3

10 30 3

10 43 3

11 27 3

11 29 3

11 39 3

12 16 3

12 18 3

12 19 3

12 25 3

12 30 3

12 31 3

12 37 3

12 39 3

12 42 3

12 44 3

12 45 3

13 15 3

13 23 3

13 24 3

13 27 3

13 29 3

14 21 3

14 22 3

14 26 3

14 39 3

15 23 3

15 24 3

15 27 3

15 29 3

15 36 3

15 37 3

16 17 3

16 18 3

16 20 3

16 23 1

16 24 1

16 27 3

16 39 3

16 42 3

16 44 3

17 16 3

17 18 3

17 21 3

17 22 3

17 26 3

17 28 3

17 30 3

17 44 3

18 16 3

18 17 3

18 19 3

18 25 3

18 30 3

18 31 3

18 36 3

18 39 3

18 44 3

19 25 3

19 30 3

19 36 3

21 32 3

21 37 3

21 39 3

21 43 3

22 26 3

22 28 3

22 36 3

22 39 3

23 33 3

23 34 1

23 35 1

23 39 3

24 27 3

24 39 3

25 29 3

25 30 3

25 39 3

26 29 3

26 39 3

27 38 3

28 38 3

29 39 3

30 31 3

30 38 3

31 30 3

31 32 3

31 33 3

31 34 2

31 35 1

31 39 3

32 31 3

32 33 3

32 34 2

32 35 1

32 39 3

33 36 3

33 37 3

33 38 3

33 39 3

34 36 3

34 37 3

34 38 3

34 39 3

35 36 3

35 37 3

35 38 3

35 39 3

36 34 3

36 35 3

36 37 3

36 38 3

36 39 3

37 33 3

37 34 2

37 39 3

37 43 2

38 39 3

38 40 3

38 45 3

39 40 3

39 44 3

40 41 3

40 44 3

40 46 3

40 47 3

40 49 3

40 50 3

41 42 3

41 44 3

41 46 3

41 47 3

41 48 3

41 49 3

41 50 3

42 43 3

42 44 3

42 46 3

42 47 3

43 37 3

43 42 3

44 45 3

44 46 3

44 47 3

44 48 3

44 49 3

45 46 3

46 47 3

46 50 3

46 51 3

47 46 3

47 48 3

47 49 3

47 50 3

47 51 3

48 46 3

48 47 3

48 50 3

48 51 3

49 46 3

49 47 3

49 50 3

49 51 3

50 51 3

50 52 3

51 52 3

52 53 3

**Supplementary Data 6. Vehicle Problem-Solving Network (‘Veh2’)**

The ‘Veh2’ problem-solving network includes 44 nodes and 249 directed links, and is represented as follows:

1 6 6

1 7 6

1 9 6

1 10 4

1 13 4

1 15 6

1 17 4

1 19 4

1 23 4

1 24 4

1 32 6

2 9 6

2 10 2

2 12 6

2 15 6

2 19 4

2 20 4

2 21 4

2 22 4

2 24 6

2 25 6

3 4 2

4 5 4

4 6 6

4 7 6

5 6 6

5 7 6

6 4 4

6 5 4

6 7 6

6 8 4

6 10 6

6 11 6

6 13 4

6 14 4

6 15 6

6 16 4

6 17 4

6 22 4

6 23 6

6 27 6

6 28 6

6 29 6

7 5 9

7 8 6

7 10 6

7 11 9

7 12 6

7 14 6

7 15 6

7 19 6

7 21 3

7 23 9

7 24 9

7 25 9

8 2 6

8 14 6

8 15 4

8 23 4

9 2 6

10 4 6

10 7 6

10 14 6

10 15 3

10 19 3

10 23 6

10 24 6

10 25 6

10 26 9

11 9 1

11 12 3

11 25 1

12 7 6

13 7 4

13 8 4

13 9 2

13 10 6

13 14 4

13 15 4

13 16 4

13 19 4

13 23 4

13 24 4

13 25 4

13 26 6

14 15 4

14 17 4

14 23 6

14 24 4

14 25 4

14 26 6

15 2 6

16 7 2

16 13 2

16 17 2

16 21 3

16 25 2

17 2 2

17 7 4

17 8 4

17 9 2

17 10 4

17 11 2

17 14 2

17 15 4

17 18 4

17 20 4

17 21 6

17 22 2

17 23 6

17 26 6

18 2 4

18 15 4

19 24 2

19 25 2

19 36 3

20 7 6

20 8 3

20 10 3

20 13 3

20 14 3

20 15 3

20 16 6

20 17 3

20 21 6

20 22 3

20 23 3

20 25 3

20 26 6

20 30 9

21 7 2

21 8 2

21 10 2

21 11 2

21 12 4

21 22 6

21 23 4

21 24 4

21 25 4

21 26 6

21 27 6

21 28 6

21 29 6

22 21 6

22 23 3

22 25 3

22 26 9

22 28 3

22 30 9

22 33 3

23 21 4

23 22 4

23 25 4

23 26 6

23 27 6

23 28 6

23 29 6

23 30 4

23 31 6

23 32 6

23 33 6

24 2 4

24 10 4

24 11 4

24 25 6

24 26 6

25 2 6

25 26 9

25 27 9

25 28 3

25 29 3

26 27 6

26 28 4

26 29 4

26 30 6

26 32 6

26 33 6

27 23 4

28 23 4

28 29 2

29 23 4

29 28 2

30 23 1

30 25 1

30 26 3

30 27 2

30 28 1

30 29 1

30 31 3

30 32 3

30 33 3

30 41 3

30 42 1

31 26 6

31 27 2

31 28 2

31 29 2

31 33 6

31 41 6

32 26 6

32 27 6

32 28 6

32 29 6

32 31 6

32 33 4

32 41 4

33 34 9

33 37 9

34 35 6

34 36 6

34 37 6

34 40 6

35 34 2

35 36 3

35 37 3

35 38 2

35 44 2

36 31 9

36 38 6

36 39 6

36 41 9

36 42 9

36 44 9

37 38 2

37 39 2

37 41 4

37 42 4

37 44 6

38 39 4

38 41 4

38 42 4

38 44 4

39 41 6

39 42 4

39 43 6

39 44 6

40 39 4

40 41 6

40 42 6

40 44 4

41 39 2

41 43 6

41 44 6

42 31 2

42 32 2

42 39 2

42 44 2

43 39 3

43 44 2

**Supplementary Data 7. Equipment Problem-Solving Network (‘Equip’)**

The ‘Equip’ problem-solving network includes 43 nodes and 120 directed links, and is represented as follows:

1 2 1

2 1 1

2 3 1

3 1 1

3 4 1

3 7 1

3 8 1

3 27 1

3 37 1

4 1 1

4 5 1

5 1 1

5 6 1

5 9 1

6 7 1

7 8 1

8 7 1

8 9 1

9 10 1

9 28 1

10 11 1

10 12 1

10 13 1

10 14 1

10 15 1

10 16 1

10 22 1

10 25 1

10 26 1

10 27 1

10 28 1

11 15 1

11 25 1

11 26 1

11 27 1

11 28 1

12 13 1

12 14 1

13 14 1

14 11 1

14 12 1

14 13 1

14 25 1

15 16 1

15 21 1

15 25 1

15 26 1

15 27 1

15 28 1

16 17 1

16 18 1

16 19 1

16 20 1

16 25 1

17 16 1

17 18 1

17 19 1

17 20 1

18 19 1

18 20 1

19 17 1

19 18 1

19 20 1

20 17 1

20 18 1

20 19 1

20 25 1

21 27 1

21 28 1

22 23 1

23 24 1

24 22 1

24 23 1

24 25 1

25 11 1

25 15 1

25 21 1

25 26 1

25 27 1

25 28 1

26 27 1

26 28 1

27 11 1

27 15 1

27 21 1

27 28 1

27 31 1

28 27 1

28 29 1

28 30 1

28 33 1

29 30 1

29 33 1

29 35 1

30 29 1

30 31 1

30 33 1

31 29 1

31 30 1

31 32 1

31 33 1

32 31 1

32 33 1

33 34 1

33 39 1

34 36 1

35 36 1

36 34 1

36 35 1

36 37 1

37 34 1

37 35 1

37 36 1

37 38 1

38 39 1

39 40 1

39 43 1

40 41 1

41 42 1

42 43 1
